# Ultrasound-assisted extraction of biosurfactants from water hyacinth for enhanced soil washing of diesel-contaminated soils: performance evaluation and phytotoxicity assessment

**DOI:** 10.1007/s11356-025-36930-2

**Published:** 2025-09-09

**Authors:** Witchaya Rongsayamanont, Naphatsarnan Phasukarratchai

**Affiliations:** Faculty of Environment and Resource Studies, Mahidol University, Salaya, Phutthamonthon, Nakhon Pathom, 73170 Thailand

**Keywords:** Water hyacinth, Biosurfactant, Soil washing, Diesel contamination, Phytotoxicity, Circular bioeconomy

## Abstract

Soil washing with surfactants is a promising technique for remediating petroleum hydrocarbon-contaminated soils. This study evaluates a biosurfactant extracted from *Eichhornia crassipes* (water hyacinth), an abundant aquatic weed in Thailand, using ultrasound-assisted extraction for diesel-contaminated soil remediation. The biosurfactant extract (Extract WH) was characterized for its surface tension reduction, critical micelle concentration (CMC), emulsification capacity with diesel, and phytotoxicity. Extract WH exhibited a CMC of 0.9443% w/v and showed enhanced emulsifying properties when combined with Dehydol LS9, as demonstrated by contact angle measurements. A surfactant formulation containing Extract WH, Dehydol LS9, and NaCl in equal proportions (1.35% w/v total) achieved 73.50% ± 5.65% diesel removal from soil contaminated at 20,000 mg/kg in 30 min. Phytotoxicity tests using rice and tomato seeds indicated improved germination rates in treated soils compared to untreated controls, although plant growth remained lower than in uncontaminated soils, possibly due to nutrient leaching. These results demonstrate the potential of water hyacinth-derived biosurfactants as an environmentally friendly and effective alternative for soil remediation.

**Graphical Abstract:**

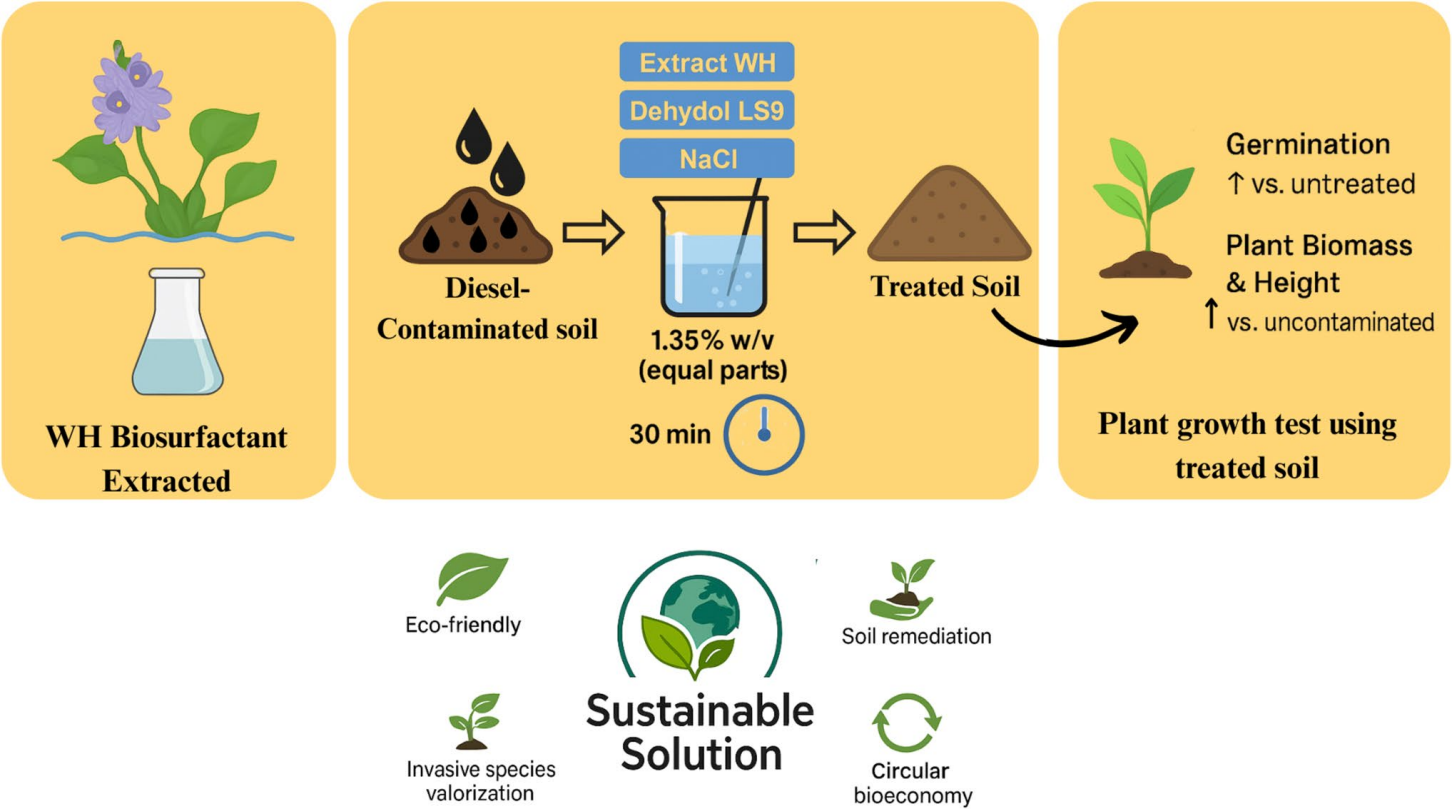

**Supplementary information:**

The online version contains supplementary material available at 10.1007/s11356-025-36930-2.

## Introduction

Water hyacinth, *Eichhornia crassipes*, is an invasive aquatic plant species widely found in freshwater bodies across Thailand. Its rapid proliferation leads to significant environmental and socio-economic problems, including water quality degradation and obstruction of waterways. According to the Department of Public Works and Town & Country Planning of Thailand, the estimated biomass of water hyacinth in Thailand exceeded 6 million tons, with its growth rate capable of doubling within 20 days (TH.DPT 2016a, b). Despite its invasive nature, water hyacinth represents a valuable biomass resource that can be utilized for bio-based product development, particularly in the extraction of natural surfactants.

Water hyacinth contains compounds with surface-active properties that can be extracted using simple ethanol-based methods (Luo et al. 2015). Previous studies have demonstrated that biosurfactants derived from water hyacinth exhibit surface tension reduction capabilities comparable to those of commercial surfactants, making them suitable for applications such as enhanced oil recovery and environmental remediation (Machale et al. 2019). Among remediation technologies, soil washing is an effective technique for the removal of petroleum hydrocarbons adsorbed onto soil particles (Bai et al. 2019; Guan et al. 2018; Peters et al. 1992). Diesel, a common fuel used in transportation, is of particular concern due to its low volatility and persistent environmental toxicity upon spillage. Diesel-contaminated soils adversely affect soil ecosystems, harming soil fauna, microbial communities, and plant growth (Banks &Schultz 2005, Safehian et al. 2018; Tang et al. 2011).

Surfactant-enhanced soil washing operates by disrupting the interfacial tension between soil particles and oil, facilitating the desorption of hydrocarbons into the aqueous phase. Surfactants possess amphiphilic molecular structures, allowing them to reduce the interfacial tension among immiscible phases such as water, oil, and soil (Rosen 2004; Tadros 2005). However, the use of synthetic surfactants can result in secondary pollution. Rongsayamanont et al. (2020) reported that nonionic surfactant mixtures were effective in diesel removal, though additional rinsing was necessary to mitigate phytotoxic effects, as evidenced by seed germination assays.

This work aims to investigate the use of biosurfactants extracted from water hyacinth for diesel-contaminated soil remediation. The research evaluates the physicochemical properties of the extracted surfactants, including surface tension, critical micelle concentration (CMC), emulsion formation with diesel, and contact angle behavior. Formulations combining natural and synthetic surfactants are also tested. Optimal washing conditions are identified through a Central Composite Rotatable Design (CCRD), considering variables such as the solid-to-liquid ratio and surfactant concentration. Post-treatment soil is assessed for phytotoxicity through seed germination tests using rice, tomato, and sunflower. The findings are expected to support the development of sustainable, bio-based surfactant applications for petroleum-contaminated soil remediation.

## Materials and methods

### Preparation of water hyacinth biomass

Fresh water hyacinth was collected from natural water sources in Nakhon Pathom Province, Thailand. The plants were washed with tap water to remove dirt and adhering contaminants, followed by rinsing with deionized (DI) water. The roots, stems, and leaves were separated and chopped into small pieces (approximately 1 cm), then naturally dried in a solar drying facility for one week. The dried biomass, containing approximately 5% moisture, was ground and sieved to obtain a fine powder passing through a 40-mesh screen (approximately 425 µm). The powdered material was kept in sealed polyethylene bags at room temperature until use.

### Extraction of surfactant from water hyacinth

Surfactant extraction was performed using aqueous ethanol solutions. Based on preliminary studies, a solvent mixture containing 75% v/v ethanol was selected to achieve optimal yield. The ethanol was prepared by diluting 95% ethanol (RCI Labscan, AR grade) with DI water. Four extraction methods were employed to compare the crude extract yields. In Methods 1, 2, and 3, 25 g of powdered water hyacinth was mixed with 250 mL of ethanol in a 500-mL Erlenmeyer flask. For Method 4, 50 g of powder was extracted with 600 mL of ethanol in a 2-L beaker. The extraction procedures for each method were as follows:

- Method 1**: **Maceration with mechanical shaking using a Wiggens orbital shaker WS-50D at 180 rpm for 120 min.
- Method 2**: **Ultrasound-assisted extraction using an Elmasonic S 60H ultrasonic water bath (150 W, 37 kHz) for 120 min.
- Method 3**: **Ultrasound-assisted extraction using an ultrasonic water bath for 60 min, followed by maceration with mechanical shaking on an orbital shaker at 180 rpm for an additional 60 min.
- Method 4**: **Ultrasound-assisted extraction using a Hielscher Ultrasonic Homogenizer UP200Ht (200 W, 26 kHz) equipped with a sonotrode S26d14 (14-mm diameter), operated at 80% amplitude for 15 min.

After extraction, the extracted solution was filtered through a Whatman No. 4 filter paper, then concentrated by using a vacuum rotary evaporator at 75 °C to remove ethanol, and dried in an oven at 60 °C for 48 h. The dried extract remaining in the flask was weighed, and the crude extract yield was calculated. The extraction method that yielded the highest amount was selected for further study.

Subsequently, the known-weight crude extract was dissolved in DI water to prepare a 10% w/v solution. This solution was stored in a glass Duran bottle at 4 °C until use. If sediment formed in the solution, only the clear supernatant was used for surfactant characterization and soil remediation experiments. The exact concentration of the crude extract solution was determined by drying a 50 mL aliquot, weighing the residue, and calculating the concentration accordingly.

### Characterization of extracted surfactant

#### Functional and chemical composition analysis

The functional groups and chemical composition of Extract WH were analyzed using FTIR and GCMS. The samples were analyzed in transmission mode using a Nicolet™ iS50 FTIR spectrometer (Thermo Fisher Scientific). The spectra were collected over a wavenumber range of 4000–400 cm⁻^1^ with a resolution of 4 cm⁻^1^ and co-added 128 times for improved signal-to-noise ratio.

The GCMS analysis was performed using the same analytical method as that employed for diesel quantification, as described in a later section. Additionally, LC-MS/MS analysis in positive ion mode was conducted using an in-house method with a Shimadzu series LCMS-8040 at the Center of Analysis for Product Quality (Division of Natural Products), Mahidol University.

#### Surface tension and CMC determination

The water-soluble fraction of the ultrasonic extract of water hyacinth obtained using Method 4, which produced the highest yield (hereafter referred to as Extract WH), was diluted at ratios ranging from 1 to 200 times the original concentration. Surface tension between the solution surface and air was measured using a tensiometer (Kino/A60) at a temperature of 25 °C. A graph was plotted to illustrate the relationship between surface tension and dilution ratio. The intersection point on the graph was used to determine the critical micellar dilution (CMD), in accordance with the surfactant theory described by Rosen (2004). The CMD value was then converted, based on the dilution factor and the original extract concentration, to obtain the CMC.

#### Phase behavior and emulsion characteristics study

The extract WH at a concentration of 1% w/v (which is higher than the CMC) and the 1% Dehydol LS9 (LS9, from Thai Ethoxylate Co., Ltd.) solution were prepared in volume ratios of 100:0, 75:25, 50:50, 25:75, and 0:100. Each 3 mL sample was mixed with an equal volume (3 mL) of diesel oil in test tubes. The height of the aqueous phase was measured before the addition of diesel oil. Deionized water (DI) was used as a control. The mixtures were then vigorously shaken, and the emulsion characteristics were observed in accordance with the surfactant theory described by Rosen (2004).

#### Contact angle determination

Contact angle measurements were determined using an optical contact angle goniometer (Kino/SL200KS) at a temperature of 25 °C, following ASTM D7334-08 method (ASTM 2022). The sessile drop method was employed, and the contact angles were calculated using the Young–Laplace fitting model. The liquid phase consisted of surfactant solutions derived from water hyacinth and LS9 in various mixing ratios, with a total concentration of 1% w/v. Three types of solid surfaces were used within the solid phase: (1) clean glass slides, (2) glass slides coated with diesel oil, and (3) Parafilm. Measurements were done in triplicate.

### Diesel-contaminated soil remediation experiment

#### Soil preparation and contamination

A total of 100 g of clean, air-dried sandy soil was mixed with 2 g of diesel oil (sourced from SUSCO gas station) and incubated for two nights to prepare diesel-contaminated soil with a target concentration of 20,000 mg/kg. This contaminated soil was used as the test matrix for soil washing experiments. For each experimental batch, contaminated soil was freshly prepared at the specified concentration and incubated for two nights in the same manner. The diesel concentration in each newly prepared soil batch was measured and referred to as Spiked soil.

#### Optimization of surfactant formulation

To determine the optimal surfactant formulation for soil washing, a mixture design approach was employed. A total of ten formulations were prepared, consisting of varying proportions of LS9, the water-soluble extract of water hyacinth (Extract WH), and NaCl (Ajax Finechem, AR grade). Each formulation had a total concentration of 2% w/v, as presented in Table 1. Each formulation was tested in duplicate. For each test, spiked soil was washed using a soil-to-solution ratio of 1.5 g of soil to 15 mL of surfactant solution in a 40-mL Clear I.Chem test tube, sealed with a PTFE-lined screw cap to prevent evaporation. The mixtures were agitated using an orbital shaker at 250 rpm for 60 min at room temperature. After shaking, the samples were allowed to settle for 1 h to facilitate phase separation, after which the supernatant was carefully decanted.

**Table 1:** Mixture design for optimal surfactant formulation for diesel soil washing

Sample run	Dehydol LS9 (%w/v)	Extract WH (%w/v)	NaCl (%w/v)
1	2.00	0.00	0.00
2	0.00	2.00	0.00
3	0.00	0.00	2.00
4	1.00	1.00	0.00
5	1.00	0.00	1.00
6	0.00	1.00	1.00
7	1.33	0.33	0.33
8	0.33	1.33	0.33
9	0.33	0.33	1.33
10	0.67	0.67	0.67

The remaining soil was subsequently washed twice with DI water at the same ratio and shaking conditions (250 rpm for 30 min per wash), allowing the soil to settle after each wash before removing the supernatant. Following the two water rinses, the washed soil was extracted and analyzed for residual diesel concentration. The soil washing efficiency was calculated based on the measured diesel concentrations in the spiked soil. The experimental data were analyzed using Statistica software to determine the optimal formulation ratio for use in subsequent experiments.

#### Determination of the optimal soil washing system

After identifying the surfactant formulation comprising Extract WH, LS9, and NaCl that yielded the highest diesel removal efficiency based on statistical analysis using the mixture design, further investigations were conducted to determine the minimum total surfactant concentration and shortest washing time that would still achieve statistically significant diesel removal efficiency from contaminated soil. This was performed using a Central Composite Rotatable Design (CCRD) for two factors, as shown in Table 2.

**Table 2:** Design of experiments using CCRD for optimization of surfactant concentration and soil washing duration

Sample run	Conc. (%w/v)	Time (min)
1	0.50	15.00
2	0.50	45.00
3	1.50	15.00
4	1.50	45.00
5	0.29	30.00
6	1.71	30.00
7	1.00	8.79
8	1.00	51.21
9 (C)	1.00	30.00
10 (C)	1.00	30.00

The experiments were carried out using spiked soil samples with the same initial diesel concentration. Soil washing was conducted at a soil-to-solution ratio of 1:10, specifically using 1.5 g of contaminated soil and 15 mL of surfactant solution. The washing process was performed with an orbital shaker at 250 rpm, followed by two rinses with deionized water, as in the procedure of Rongsayamanont et al. (2020), prior to diesel extraction and analysis of the residual diesel content. The washing efficiency was calculated, and the optimal surfactant concentration and washing duration were determined using the desirability function in CCRD analysis, conducted with Statistica software. Subsequently, soil washing was performed using the optimal conditions predicted by the statistical model in order to evaluate the deviation between the experimental results and the predicted values.

#### Scale-up of soil washing for preparation of plant growth test soil

Based on the optimal soil washing conditions identified through statistical analysis and validated by experimental trials in test tubes using an orbital shaker, the most effective formulation was found to be a total concentration of 1.35% w/v, including Extract WH, LS9, and NaCl in a ratio of 1:1:1. Soil washing was conducted with shaking at 250 rpm for 30 min, using a soil-to-solution ratio of 1 g:10 mL.

For scale-up, the washing process was carried out in a 2 L beaker using 100 g of diesel-contaminated soil and 1 L of surfactant solution. Mixing was performed using an overhead stirrer (DragonLab Model OS20-Pro) equipped with a four-blade propeller stirrer (Propeller No. 18900071) with a diameter of 5 cm. The mixing speed was maintained at 750 rpm for 30 min. The propeller was positioned 1.5 cm above the bottom of the beaker, and the stirrer shaft was centered within the beaker. After mixing, the slurry was allowed to settle for 1 h. The soil was then rinsed twice with deionized water under the same stirring speed and duration. After the final rinse, the soil was collected for extraction and further chemical analysis.

For soil washing intended for plant growth testing, 200 g of sandy soil was spiked with diesel fuel at a concentration of 20,000 mg/kg and incubated for two nights. The contaminated soil was then washed in two separate batches using the same optimized conditions and subsequently combined for use in planting experiments in a single pot.

### Plant growth test using washed soil

Uncontaminated soil, diesel-contaminated soil, and diesel-contaminated soil that had undergone the washing process were each used at a quantity of 200 g per pot for plant growth testing. Three plant species were selected: rice, tomato, and sunflower. For rice and sunflower, 35 seeds were sown per pot, while 40 seeds were sown for tomato. Rice seeds were soaked in water overnight, and only seeds that sank and showed a white germination point were selected for planting. Sunflower and tomato seeds were commercially packaged seeds from Chia Tai Co., Ltd. and were used directly without any soaking or washing processes. A total of nine pots were used in the experiment.

On day 0 of planting, each pot was watered with 50 mL of water. All pots were placed in trays with lids to retain moisture and kept in a dark environment for 10 days. After this period, the lids were removed, and the pots were exposed to morning light daily. Germination counts were recorded every 10 days for a total of 30 days for tomato and sunflower, and 20 days for rice. At the end of the respective observation periods, plants were harvested for growth measurements. Plant height was measured from the soil surface to the tip of the tallest shoot. For rice, the height was recorded up to the tallest leaf tip. Plants were weighed to determine biomass. Due to the small mass of rice and tomato seedlings, the total weight per pot was measured and averaged per number of germinated seedlings. Sunflower seedlings were individually weighed.

The seed germination percentage was calculated based on the number of seeds that germinated relative to the total number sown. The relative germination percentage was also determined as a percentage of the germination observed in the control sample (uncontaminated soil). In addition, shoot height, relative shoot height, average seedling weight, and relative average weight were calculated.

Post-harvest soil samples were extracted and analyzed for residual diesel content, soil texture, total organic carbon (TOC), total nitrogen (TN) using a TOC/TN analyzer, organic matter (OM), available phosphorus (P), and potassium (K). These soil properties were assessed to support the interpretation of plant growth outcomes.

### Diesel quantification by GC-MS

Diesel-contaminated soil samples that had undergone laboratory-scale soil washing (from an initial weight of 1.5 g), along with 1.5 g of spiked soil samples, were extracted using 20 mL of dichloromethane (QRëC, AR grade) mixed with 5 mL of 95% ethanol. The extraction was carried out in tightly sealed test tubes to prevent volatilization, followed by shaking at 250 rpm for 4 h at room temperature. A 1-mL aliquot of the extract was then drawn using a syringe and filtered through a 0.45-μm PTFE syringe filter into a sample vial for subsequent analysis by gas chromatography–mass spectrometry (GC-MS) using a Shimadzu TQ8040 instrument. The analysis was performed using an Rxi-5Sil MS capillary column (30 m × 0.25 mm I.D., 0.25 μm film thickness). One μL of the sample was injected with helium as the carrier gas at a pressure of 100 kPa. The total flow rate was 20 mL/min, with a column flow rate of 1.78 mL/min. The column temperature program followed the diesel analysis method described by Rongsayamanont et al. (2020), starting at 40 °C (held for 5 min), then ramped at 15 °C/min to 300 °C, and held for 5 min. The injector temperature was maintained at 300 °C. For the mass spectrometer, the ion source and interface temperatures were set at 200 °C and 300 °C, respectively. In addition, the compound composition in Extract WH was analyzed using the same GC-MS conditions, and the resulting spectra were compared against a compound database library to identify similar compounds.

For soil samples washed at a larger scale in beakers, after separating the supernatant, 1.5 g of soil was weighed and extracted following the same procedure as above. To determine the moisture content of these samples, a separate 30-g soil sample was placed in a pre-weighed beaker and dried at 103 °C. The moisture data were used to adjust the extraction results, allowing for an accurate comparison of diesel removal efficiency relative to the spiked samples.

## Results and discussion

### Crude extract yields and method efficiency in surfactant recovery from water hyacinth

**Fig. 1:**
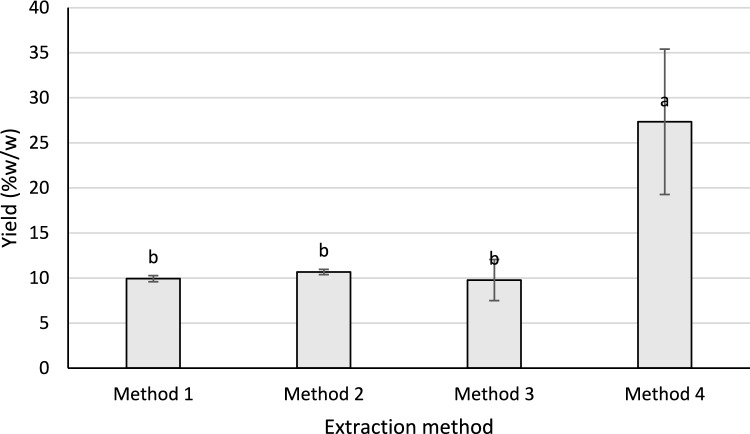
Yield of crude Extract WH obtained from different extraction methods. a and b indicate significant differences by one-way ANOVA at *p* = 0.05

This study investigated the efficiency of different extraction methods for isolating biosurfactants from water hyacinth and evaluated their initial physicochemical properties. Among the four tested methods—(1) flask shaking, (2) ultrasonic bath, (3) combined shaking and ultrasonic bath, and (4) direct ultrasonic probe—the highest extraction yield was achieved using Method 4, which applied direct ultrasonic probe treatment. This method produced a yield of 27.3% (Fig. 1). In contrast, Methods 1, 2, and 3 yielded lower amounts of extract and showed no statistically significant difference among them, as indicated by the ANOVA result (*p* = 0.05).

The high efficiency of Method 4 was consistent with the mechanism described by Zahari et al. (2020), where ultrasonic waves directly disrupt plant cell walls, facilitating greater solvent penetration and solute release. In contrast, the ultrasonic bath (Method 2) and the combined shaking with ultrasonic bath (Method 3) were less effective. This limitation was likely due to the indirect application of ultrasonic energy in the bath system, where the energy must pass through water before reaching the extraction vessel, thereby reducing the intensity of cavitation on plant material. This is similar to the findings of Ranjha et al. (2021), which indicated that indirect sonication is less efficient than direct sonication for biomass extraction.

Paredes Selva Filho A.A. (2020) reported the presence of saponins in water hyacinth root extracts, which exhibit emulsifying properties for petroleum-based oils, including diesel. Their study also demonstrated over 60% efficiency in washing oil-contaminated sandy soils. Additionally, Raza and Gates (2021) showed that nanocellulose-based solutions can significantly reduce interfacial tension between water and mineral oils by enhancing oil dispersion. These findings suggest that the direct ultrasonic probe method may have extracted higher levels of functional biomolecules, such as cellulose and saponins, contributing to improved surfactant properties in Extract WH.

The crude extract obtained via direct ultrasonic probe appeared as a greenish-brown viscous substance. Due to its partial insolubility in water, only the fully water-soluble fraction was used for further applications. This fraction, referred to as Extract WH, had a concentration of 4.72 ± 0.02% w/v after filtration, drying, and redissolution in DI water.

### Functional and chemical composition of Extract WH

#### FTIR analysis

**Fig. 2:**
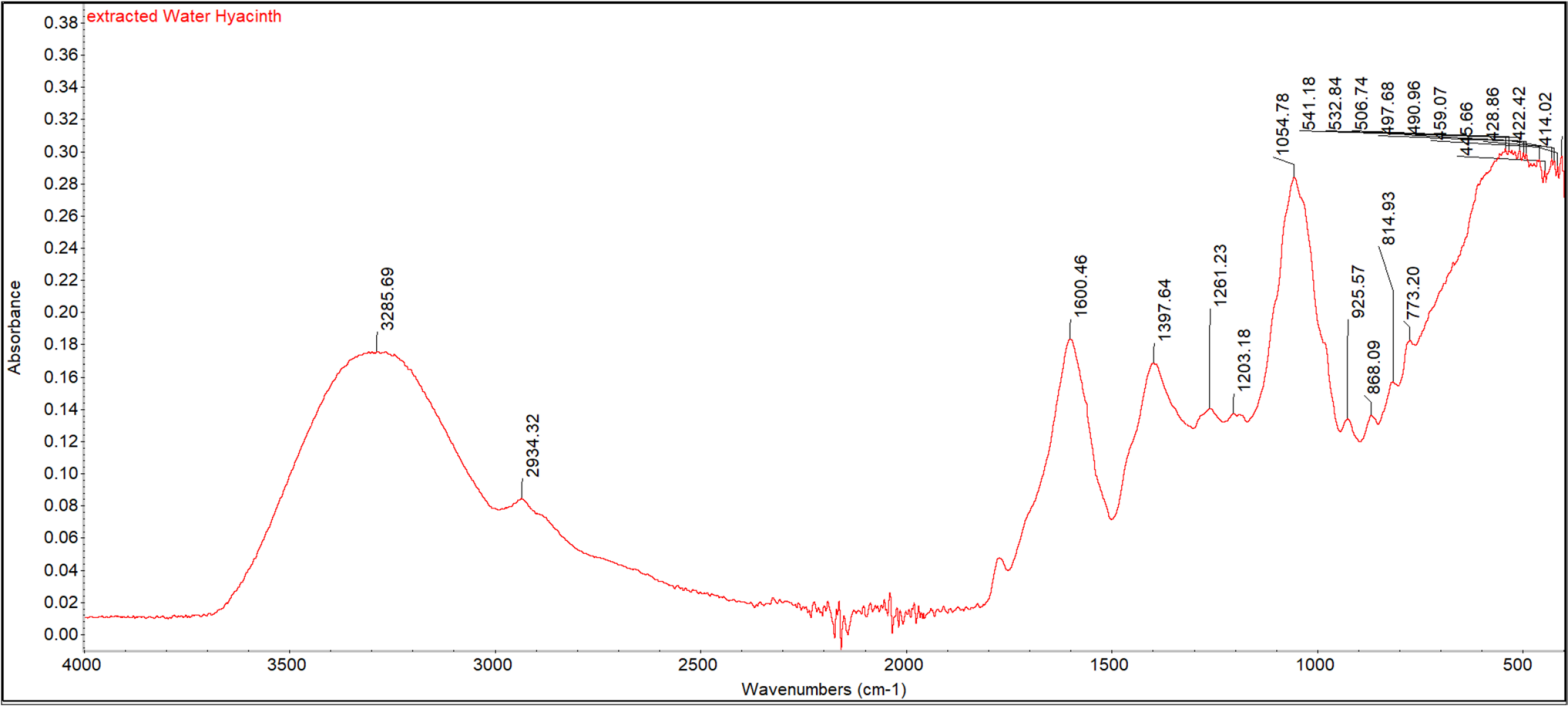
FTIR absorption spectra in the range of 4000–400 cm⁻^1^ of water hyacinth extract obtained by ultrasonic extraction

The functional groups of compounds present in the water hyacinth extract obtained via direct ultrasonic extraction were analyzed following the removal of ethanol and complete drying of residual water. FTIR was employed to characterize these compounds, with absorption spectra recorded over the range of 4000–400 cm⁻^1^, as illustrated in Fig. 2. The FTIR analysis revealed characteristic peaks at wavenumbers 3285.69, 2934.32, 1600.46, 1397.64, and 1054.78 cm⁻^1^. These were assigned to O–H stretching (hydrogen bonding with hydroxyl groups), C–H stretching of aliphatic saturated components, C = O stretching of esters and aldehydes associated with hemicellulose and lignin bands, vibrations of CH_2_ groups typical in crystalline cellulose, and C–O stretching of cellulose, hemicellulose, or lignin, respectively. The FTIR chromatogram showed functional group absorption patterns similar to water hyacinth cellulose fibers, aligning with the findings of Ewnetu Sahlie et al. (2022), George et al. (2023), and Rezania et al. (2018). This comparison provided insights into variations in solubility within the extract.

#### GCMS analysis

The GCMS analysis of Extract WH, derived from water hyacinth via direct ultrasonic probe-assisted extraction, identified a broad range of phytochemical constituents with potential surface-active properties. The mass spectra of the compounds were compared with reference libraries (in Supplementary Information Fig.S1-20) and the corresponding chromatogram. Identified compounds were summarized in Table 3. These results reflected a chemically diverse profile that may explain the extract’s observed surfactant-like behavior.

**Table 3:** Major phytochemical compounds identified in Extract WH by GCMS

Name	Retention time (min)	m/z	Area (%)	Height (%)
2-Propenoic acid, 2-methyl-, 2-methylpropyl ester	8.153	69	5.71	2.84
1-Butanol, 3-methyl-, formate	10.322	55	3.69	2.48
1H-Pyrrole-2,5-dione, 3-ethyl-4-methyl-	11.878	139	3.30	4.00
Benzeneacetic acid	11.99	91	4.72	3.63
Benzaldehyde, 4-hydroxy-	13.115	121	2.11	1.91
Vanillin	13.499	151	5.70	6.93
2(4H)-Benzofuranone, 5,6,7,7a-tetrahydro-4,4,7a-trimethyl-, (R)-	14.734	111	3.44	4.03
Fumaric acid, ethyl 2-methylallyl ester	14.92	127	29.51	34.95
Diethyl phthalate	15.104	149	5.95	4.52
3-Hydroxy-.beta.-damascone	15.319	69	2.07	2.41
4-Hydroxy-.beta.-ionone	15.392	109	0.80	0.84
2-Cyclohexen-1-one, 4-(3-hydroxy-1-butenyl)−3,5,5-trimethyl-	15.572	108	9.39	9.58
3-Buten-2-one, 4-(4-hydroxy-2,2,6-trimethyl-7-oxabicyclo[4.1.0]hept-1-yl)-	15.895	123	4.95	5.63
6-Hydroxy-4,4,7a-trimethyl-5,6,7,7a-tetrahydrobenzofuran-2(4H)-one	16.337	178	1.61	1.65
6-Hydroxy-4,4,7a-trimethyl-5,6,7,7a-tetrahydrobenzofuran-2(4H)-one	16.564	111	7.44	8.24
2-Cyclohexen-1-one, 4-hydroxy-3,5,6-trimethyl-4-(3-oxo-1-butenyl)-	16.637	124	3.93	2.55
n-Hexadecanoic acid	17.779	73	1.76	1.49
1,1':3',1''-Terphenyl, 5'-phenyl-	23.56	306	2.05	1.52
Stigmasterol	25.853	55	1.85	0.79

The dominant compound in the extract was *fumaric acid, ethyl 2-methylallyl ester*, with the highest relative area (29.51%) and peak height (34.95%) at a retention time (RT) of 14.92 min. Although not a typical biosurfactant, fumaric acid esters can be used as biocides (NIH 2025f) and might exhibit interfacial activity under specific conditions, particularly in emulsification or solubilization of hydrophobic compounds.

Another notable compound was *2-cyclohexen-1-one, 4-(3-hydroxy-1-butenyl)−3,5,5-trimethyl-* (RT: 15.572 min; 9.39% area) or 3-Oxo-alpha-ionol, which contains both hydroxy and ketone functional groups (NIH 2025h). These polar groups may influence the extract’s solubility profile and polarity, contributing to amphiphilic behavior.

*Diethyl phthalate* (RT: 15.104 min; 5.95% area) is commonly regarded as a contaminant from plastic sources (NIH 2025g). This shows that the natural source of this water hyacinth was contaminated with plastic waste.

*Vanillin* (RT: 13.499 min; 5.70% area), an aromatic aldehyde with known antioxidant properties, which may participate in chemical interactions with both polar and nonpolar compounds due to its phenolic structure.

Additionally, *2-propenoic acid, 2-methyl-, 2-methylpropyl ester* (RT: 8.153 min; 5.71% area) or isobutyl methacrylate is an unsaturated ester (NIH 2025e), which may contribute to interfacial modification, although it is not a biosurfactant by definition.

Compounds such as *n-hexadecanoic acid* (palmitic acid, RT: 17.779 min; 1.76% area) and *stigmasterol* (RT: 25.853 min; 1.85% area) are characteristic of plant-derived surfactants. Palmitic acid is a saturated fatty acid often associated with anionic surfactant behavior (Alves Teixeira da Rocha et al. 2024). Stigmasterol, a phytosterol, is lipophilic and associated with plant saponins, which function as natural surfactants (Srivastava &Shukla 2003).

Collectively, the presence of esters, oxygenated hydrocarbons, fatty acids, aromatic aldehydes, and plant sterols points to a mixture capable of weak to moderate surface activity. Although classical biosurfactants such as rhamnolipids or sophorolipids were not detected, the chemical composition suggests potential for amphiphilic interactions. Oxygen-containing functional groups (hydroxyl, ester, and carbonyl) may enable interactions with both hydrophilic and hydrophobic phases, possibly contributing to emulsification and surface tension reduction.

The chemical composition of Extract WH was consistent with earlier reports. Additionally, the GCMS profile showed similarities to ethanol extracts of water hyacinth studied by Okwadha and Makomele (2018), particularly in the presence of saponin-like and fatty acid components.

#### LCMSMS analysis

The LCMSMS analysis in positive ionization mode of Extract WH revealed the presence of several amphiphilic compounds with potential surfactant-like behavior. The mass spectral data indicated a range of molecular species that may contribute to the surface activity observed in the extract’s physicochemical assays (in Supplementary Information section 2).

## 1) Fatty acid and amide derivatives

One of the most prominent compounds identified was *ricinoleic acid* (C_18_H_34_O_3_) at *m/z* 299.1831 with a RT of 14.0 min. Ricinoleic acid is a hydroxylated long-chain fatty acid known for its amphiphilic nature, arising from the combination of a hydrophobic alkyl chain and a polar hydroxyl group. It is frequently utilized in industrial surfactants and emulsifying agents due to its surface-active properties (NIH 2025d). Two fatty acid amides were also detected: *palmitic amide* (C_16_H_33_NO) at *m/z* 256.2644 (RT: 19.4–20.8 min) and *stearamide* (C_18_H_37_NO) at *m/z* 284.2953 (RT: ~ 21.2–21.3 min). These compounds are structurally composed of long hydrophobic chains and polar amide groups, enabling them to orient at interfaces and function as nonionic surfactants. Such amides are commonly included in cosmetic formulations and industrial emulsifiers (NIH 2025b, c).

## 2) Amphiphiliclipid compounds

Another compound of interest is *alpha-tocotrienol* (C_29_H_44_O_2_), detected at *m/z* 425.2876–425.2879 with a RT of approximately 19.5–19.6 min. A derivative of vitamin E, alpha-tocotrienol contains a polar chromanol head and a hydrophobic isoprenoid tail. Although its primary role is antioxidant, recent studies have shown its potential involvement in membrane interactions and emulsion stabilization, especially in lipid-based delivery systems (Mohamad 2023, Woollard &Indyk 2003).

## 3) Saponin derivatives

A significant natural surfactant detected was *Cimiracemoside* D (C_37_H₅_8_O_11_) at *m/z* 679.5133 and a RT of 9.2–9.3 min. Cimiracemoside D is a saponin glycoside including both hydrophobic triterpenoid aglycones and hydrophilic sugar moieties(NIH 2025a). Such molecular architecture enables saponins to reduce surface tension, form stable foams, and act as emulsifiers in aqueous systems (Sparg et al. 2004).

The LCMSMS findings confirmed that Extract WH contains several amphiphilic compounds, including hydroxylated fatty acids (ricinoleic acid), fatty acid amides (palmitic amide and stearamide), vitamin E analogues (alpha-tocotrienol), and glycosidic saponins (Cimiracemoside D). These compounds collectively contribute to the extract’s ability to lower surface tension and form emulsions, as observed in laboratory performance tests. Their presence substantiates the hypothesis that Extract WH possesses natural surfactant properties. These results validate its suitability for application in environmental remediation, particularly in washing or treating diesel-contaminated soils, supporting both its efficacy and green chemistry potential.

## Surfactant characterization of Extract WH

### Surface tension reduction capability

**Fig. 3:**
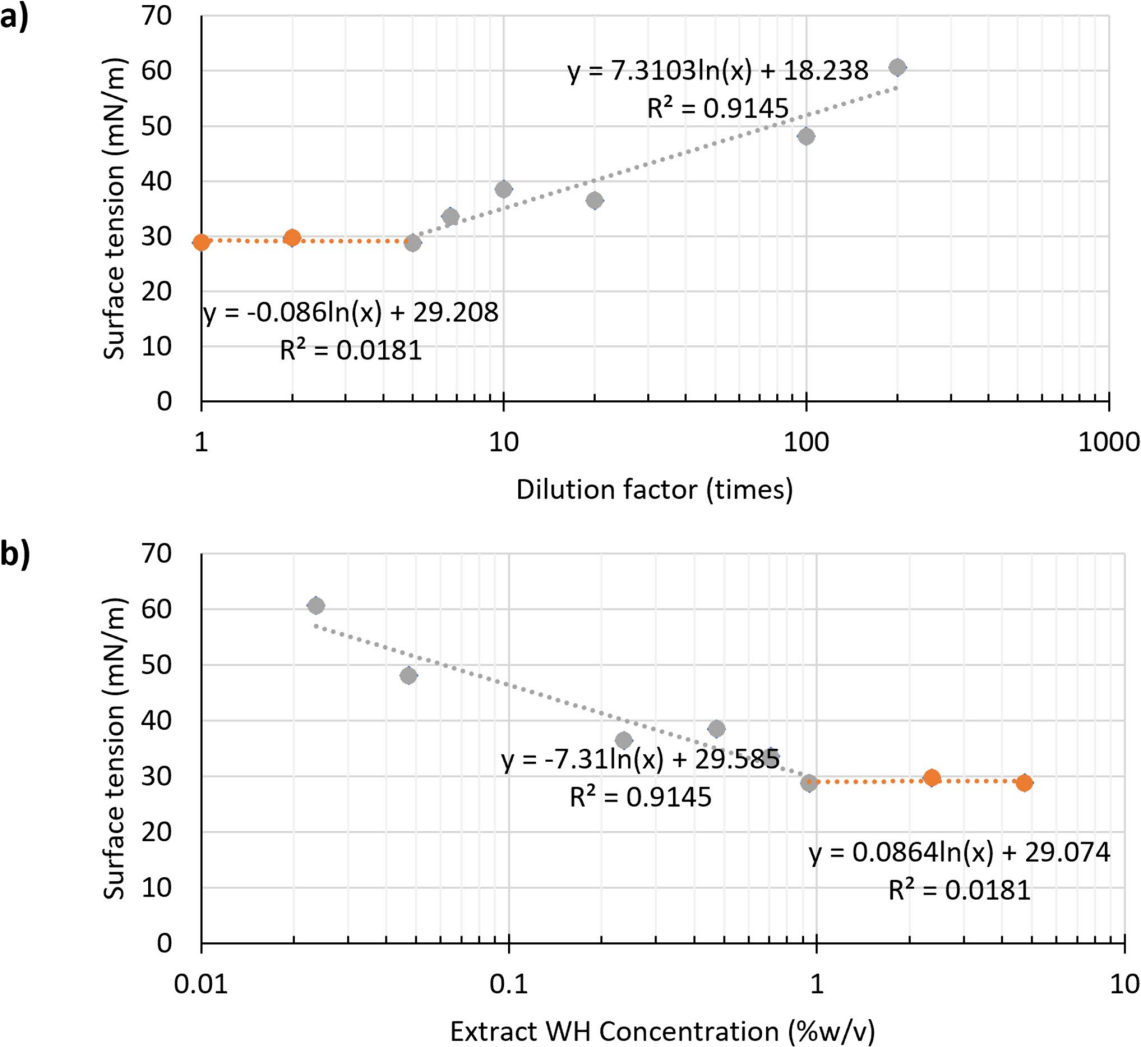
Surface tension at the air–liquid interface of water hyacinth extract (Extract WH): **a** surface tension values in relation to dilution from the concentrated extract and **b** relationship between extract concentration and surface tension indicating the critical micelle concentration (CMC)

Extract WH demonstrated the ability to reduce the surface tension at the air–water interface. When the aqueous extract was diluted within the range of one- to fivefold, the surface tension decreased from the typical value of pure water at 72 mN/m to a minimum of 29.14 ± 0.52 mN/m. As dilution increased beyond this range, the surface tension values gradually rose, indicating a concentration-dependent effect, as illustrated in Fig. 3a. Further analysis of the relationship between extract concentration and surface tension (Fig. 3b) revealed that the surface tension remained relatively constant at concentrations equal to or greater than 0.9443% (w/v). This trend is characteristic of surfactant behavior, in which the addition of amphiphilic molecules to water leads to a progressive decrease in surface tension up to a critical threshold, after which further additions have minimal impact. This threshold corresponds to the CMC at which micelle formation begins and surface tension plateaus. The observed surface activity thus confirms that Extract WH functions effectively as a biosurfactant. The measured CMC of 0.9443% (w/v) for Extract WH indicated the point of saturation beyond which no further reduction in surface tension occurs, signifying efficient micelle formation and interfacial stabilization. Compared to Selva Filho et al. (2021), our extract exhibits a similar surface tension but has a CMC that is approximately 6.75 times higher (0.14%w/v).

### Phase behavior of Extract WH and LS9 mixtures with diesel

**Fig. 4:**
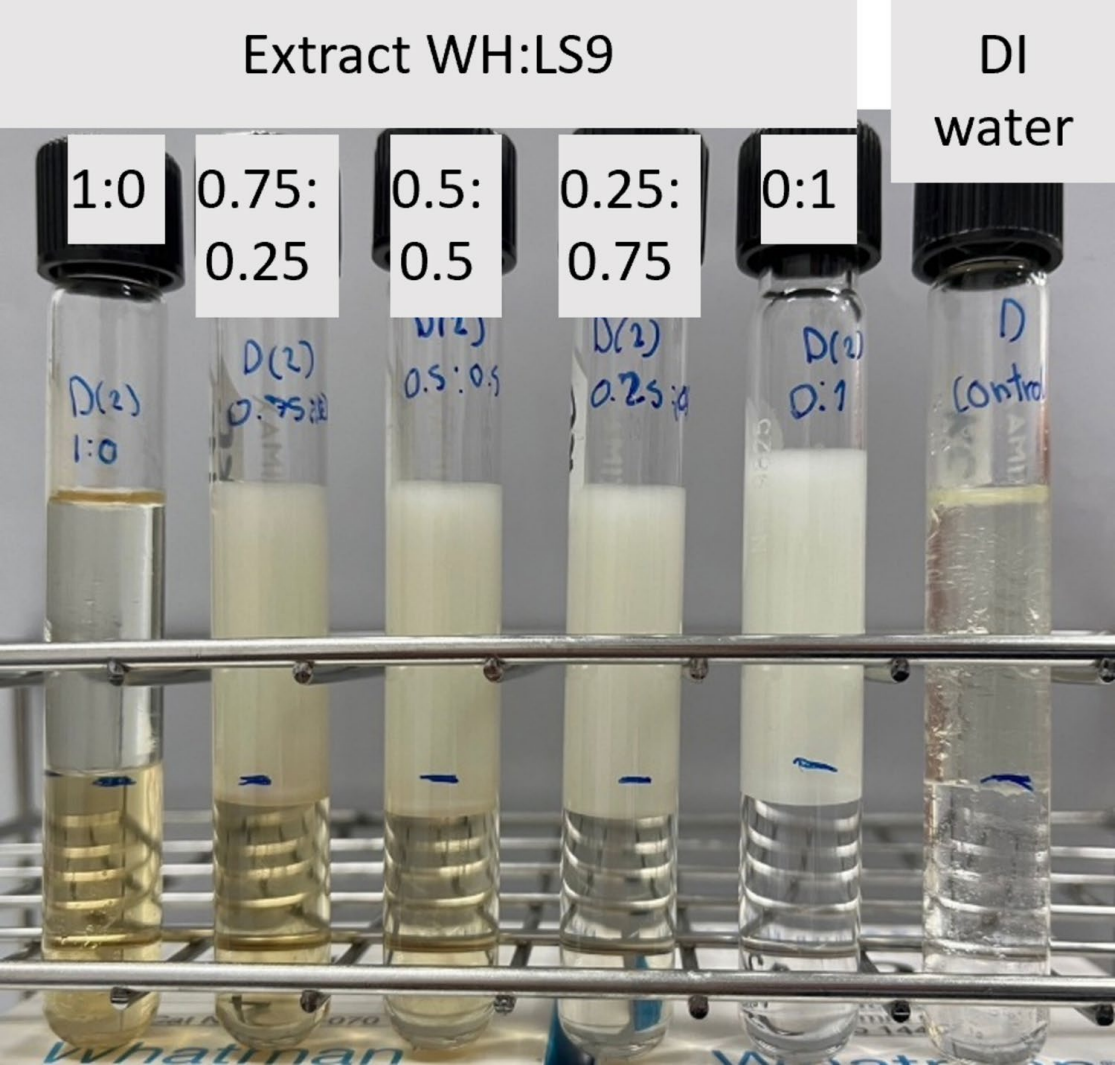
Phase behavior and emulsion characteristics of diesel mixed with various ratios of Extract WH and LS9 at a total surfactant concentration of 1% w/v. The control sample contains only DI water and diesel

The phase behavior of surfactant solutions in contact with diesel was investigated using binary mixtures of Extract WH and the commercial surfactant, Dehydol LS9 (LS9) at a total concentration of 1% w/v. As illustrated in Fig. 4, when Extract WH was used alone (far-left bottle), no noticeable change in the water level at the interface with diesel oil was observed. This suggests limited interaction or emulsification capacity under these conditions. However, when LS9 was incorporated into the mixture, distinct changes were observed at the water–diesel interface. The diesel phase floating above the aqueous layer became visibly turbid, indicating the formation of a water-in-oil (W/O) emulsion. The degree of emulsion formation increased with higher proportions of LS9, suggesting enhanced surfactant performance when LS9 is present.

From visual inspection, the emulsification patterns of mixtures with Extract WH:LS9 ratios of 0.5:0.5 and 0.25:0.75 closely resembled that of the 0:1 ratio (LS9 only), demonstrating that the emulsifying behavior was strongly influenced by the presence and proportion of LS9. This behavior confirmed that the combination of Extract WH with a synthetic surfactant such as LS9 improves the ability to emulsify diesel oil, with greater efficacy as the LS9 content increases.

### Contact angle behavior of Extract WH and LS9 mixtures on various surfaces

**Fig. 5:**
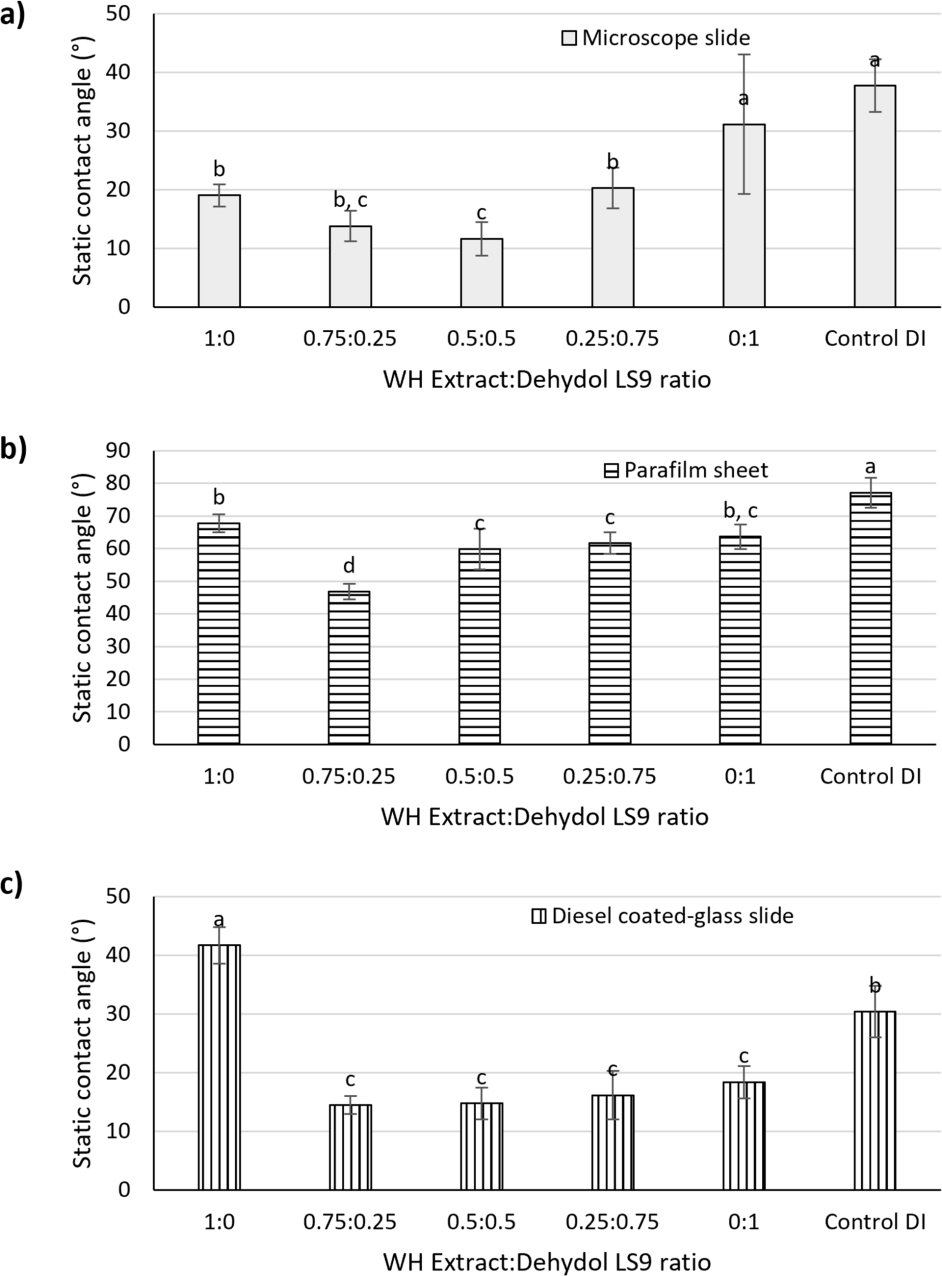
Average contact angles of 1% w/v surfactant solutions with varying ratios of Extract WH and Dehydol LS9 measured on three solid surfaces: **a** glass microscope slide, **b** parafilm, and **c** diesel-coated glass slide. Here a, b, c, and d indicate significant differences by one-way ANOVA at *p* = 0.05 for each figure

Contact angle measurements were conducted to assess the wettability of mixed surfactant solutions comprising Extract WH and LS9, prepared at a total concentration of 1% w/v with varying ratios. These were compared against DI water on three different solid surfaces: (a) microscope glass slide (Fig. 5a), (b) parafilm (Fig. 5b), and (c) diesel-coated glass slide (Fig. 5c). The results revealed that the mixture with an Extract WH:LS9 ratio of 0.75:0.25 exhibited the lowest contact angle across parafilm and diesel-coated surfaces. Overall, the combination of Extract WH and LS9 demonstrated superior wettability compared to each component individually. This was consistent with literature findings indicating that mixed surfactant systems often perform better than single surfactants in terms of spreading, emulsification, and interfacial behavior (Kumar Shah et al. 2022). The addition of LS9 consistently reduced the contact angle relative to Extract WH alone, indicating improved surface affinity and film formation—particularly on the hydrophobic diesel-coated glass. This implies that the combination of Extract WH with LS9 synergistically enhanced surface activity, likely improving interfacial tension (IFT) reduction between water and diesel.

Despite these promising results, contact angle measurements alone were insufficient to determine the optimal surfactant ratio for diesel-contaminated soil washing. Soil washing involves complex multiphase interactions among soil particles, diesel, and water, requiring surfactants to effectively reduce interfacial tensions across all involved phases. Therefore, additional soil washing experiments were necessary to evaluate the performance of different surfactant mixtures under realistic conditions and to identify the most effective formulation.

## Soil washing

### Optimization of surfactant mixture ratios of Extract WH, LS9, and NaCl

To determine the optimal mixing ratios among LS9, Extract WH, and NaCl, a mixture design experiment was conducted using a simplex-lattice design with interior points, including the overall centroid. The experiment evaluated diesel-contaminated soil with an initial concentration of 20,000 mg/kg using surfactant mixtures at a total concentration of 2% w/v, covering 10 formulations with duplicates each. All surfactant formulations performed better than deionized water alone in terms of diesel removal efficiency. Statistical analysis indicated that the experimental data were best described by a full cubic regression model, which exhibited the highest values for both *R*^2^ and adjusted *R*^2^. The lack of fit test was not statistically significant (*p* = 0.8924), confirming that the regression model is suitable for predicting removal efficiency within the experimental range. Detailed statistical results and the predictive model equation are provided in the Supplementary Information (Section 3).

**Fig. 6:**
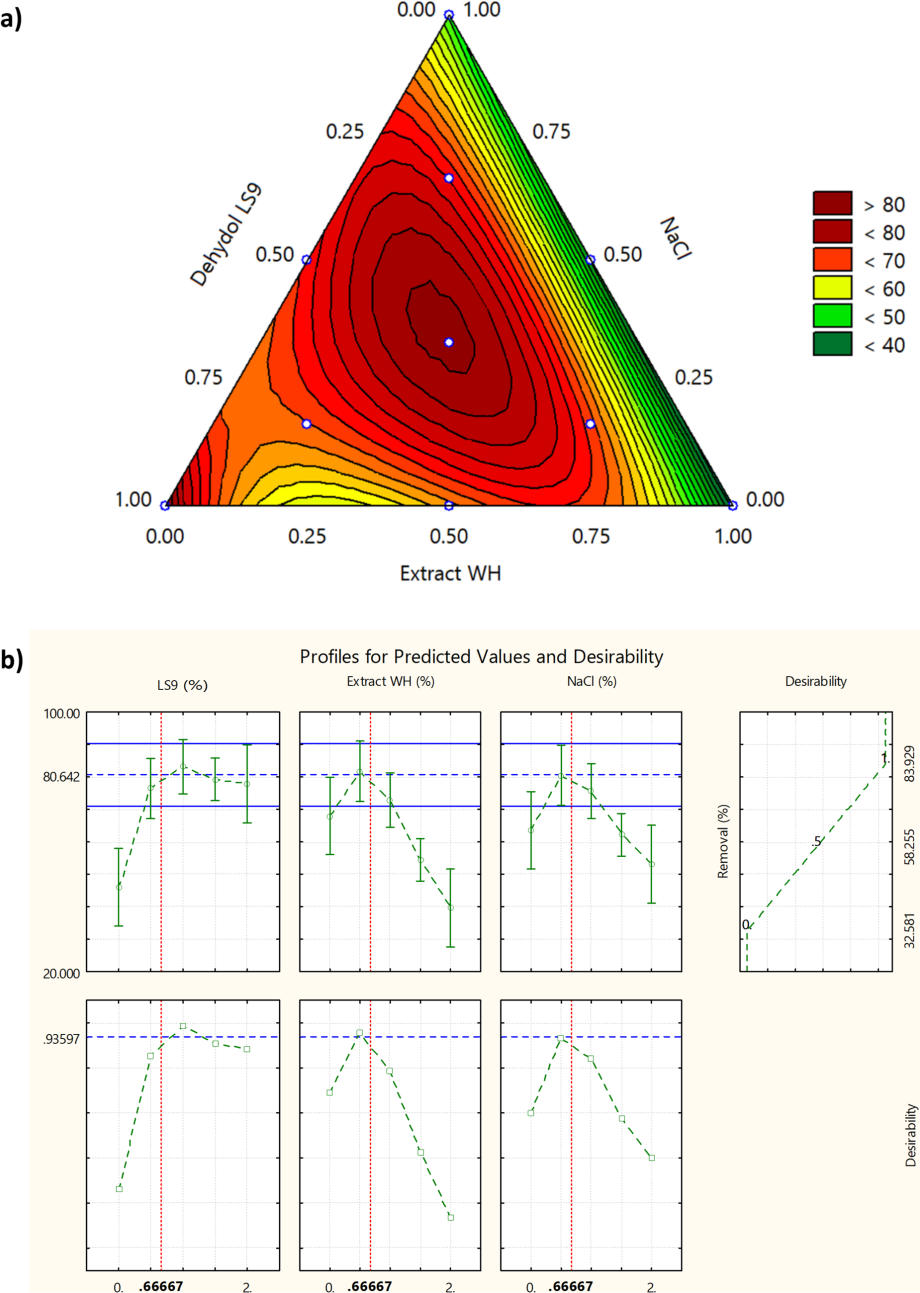
Diesel removal efficiency using surfactant mixtures of Extract WH, Dehydol LS9, and NaCl at a total concentration of 2% w/v: **a** ternary contour plot showing removal performance across different mixture ratios. **b** Predicted response profiles and desirability analysis indicating the optimal formulation

As illustrated in Fig. 6a, all three components—Extract WH, LS9, and NaCl—significantly influenced diesel removal efficiency at the 2% w/v total concentration level (*p* < 0.05). The strongest positive effect was observed with increasing proportions of LS9, a nonionic surfactant. However, when Extract WH exceeded 1%, a decline in performance was noted. Similarly, increased NaCl concentration led to reduced efficiency, indicating a nonlinear effect. A formulation containing equal proportions (1:1:1) of Extract WH, LS9, and NaCl was identified as the optimal combination based on desirability analysis, achieving a predicted removal efficiency of 80.64% (Fig. 6b). This prediction was validated experimentally, with actual removal reaching 80.94%, reflecting a prediction error of 0.37%.

In surfactant-based soil washing systems, the addition of salts or electrolytes can enhance removal efficiency via the salting-out effect, which promotes micelle formation and improves the solubilization of non-polar organic compounds. This effect is particularly pronounced for anionic surfactants (Beunen &Ruckenstein 1982, El Haber et al. 2023). Extract WH, derived from water hyacinth, contains various plant-based compounds, including fatty acids as confirmed by GCMS and LCMSMS analysis. These fatty acids act as anionic surfactant-like molecules, and the addition of NaCl may improve their solubilizing capacity. However, this enhancement occurs only up to an optimal salt concentration. Excessive NaCl can lead to charge neutralization between the anionic surfactant and the salt’s cations, resulting in precipitation or reduced solubility (Rosen 2004). This may help explain the observed decline in efficiency with higher NaCl content in the surfactant mixture.

Based on these results, the equal-ratio formulation (1:1:1) of LS9, Extract WH, and NaCl was selected for further optimization studies focused on extraction time and surfactant concentration, to enhance the soil washing process for diesel-contaminated environments.

### Optimization of surfactant concentration and washing time

In soil washing processes, prolonged treatment time may lead to unnecessary energy consumption and increased operational costs. Moreover, extended contact time can enhance surfactant adsorption onto soil particles, which in turn reduces the availability of dissolved surfactant in the aqueous phase for effective diesel solubilization. Consequently, identifying an optimal washing time that balances maximum removal efficiency with minimal energy use is crucial. Similarly, it is essential to determine the lowest surfactant concentration that still achieves statistically significant diesel removal.

**Fig. 7:**
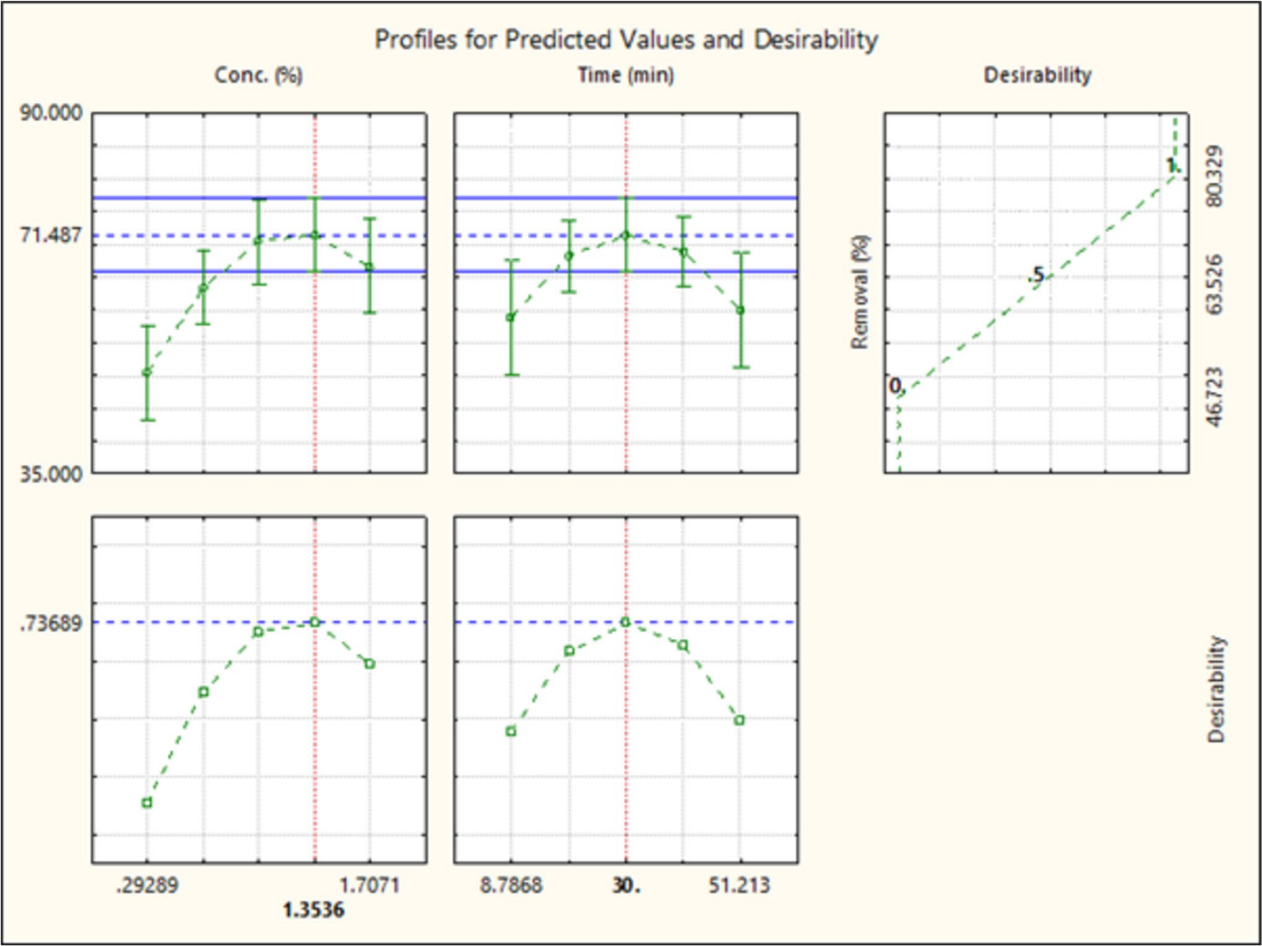
Desirability analysis of optimal surfactant concentration and washing time for diesel-contaminated soil by CCRD

To optimize the surfactant concentration and washing time, a mixture formulation with a fixed ratio of Extract WH:LS9:NaCl at 1:1:1 was evaluated using CCRD. Statistical analysis using desirability profiling revealed that increasing surfactant concentration significantly enhanced diesel removal efficiency (Fig. 7). Additionally, extending the washing time improved removal performance up to a certain point—30 min. Beyond this duration, removal efficiency began to decline. The soil-to-liquid ratio was maintained at 1:10 g/mL throughout the experiments. The optimal conditions determined by desirability analysis were a total surfactant concentration of 1.35% w/v and a washing time of 30 min, predicting a diesel removal efficiency of 71.49%. This condition was selected for further scale-up testing. These statistical results are provided in the Supplementary Information (Sect. 4).

Following the identification of the optimal formulation, washing time, and concentration, a scale-up experiment was conducted. The lab-scale system initially used 1.5 g of soil with 15 mL of solution, agitated at 250 rpm. For the scale-up, 100 g of soil and 1 L of surfactant solution were mixed in a 2-L beaker using an overhead stirrer set to 750 rpm—a speed sufficient to homogenize without causing excessive foaming. The soil was washed for 30 min, followed by two rinses with clean water, each lasting 30 min. The scaled-up system achieved a diesel removal efficiency of 73.50% ± 5.65%, which closely matched the predicted value with only a 2.74% deviation. These results indicated that the optimized conditions are applicable to larger-scale operations and can be feasibly transferred from laboratory to practical implementation. The treated soil from this process was used in subsequent plant growth experiments to assess its suitability for reuse.

In the study by Selva Filho et al. (2021), the application of water hyacinth extract for the remediation of motor oil-contaminated sand achieved removal efficiencies ranging from 66.25% to 67.50%, when using extract concentrations from 0.5 × to 2 × the critical micelle concentration (CMC). In contrast, the present study found that applying approximately 2 × CMC of our extract resulted in only 36% diesel removal from contaminated sand. However, when using a biosurfactant mixture derived from the extract, the removal efficiency increased significantly, reaching up to 70%. When compared to the findings of Whang et al. (2008), which utilized biosurfactants such as rhamnolipid and surfactin to enhance diesel biodegradation, a significantly higher removal efficiency was observed. Specifically, the researchers reported that 80 mg/L of rhamnolipid could achieve complete (100%) diesel degradation. In contrast, this study employed a physical washing method using a biosurfactant mixture at 1.35% w/v, which achieved 73.50% ± 5.65% diesel removal within 30 min—a result considered highly efficient under controlled laboratory conditions. Furthermore, Musa et al. (2020) investigated the use of fresh and dried water hyacinth for remediating diesel-contaminated soil, reporting that the application of 100 g of fresh water hyacinth to 2 kg of soil reduced total petroleum hydrocarbons by up to 59.7% over a 90-day period. These findings reinforce the potential of water hyacinth as an effective bioremediation material. However, the use of its water-soluble extract, as demonstrated in this study, offers a faster and more controllable approach to soil remediation, particularly under short treatment durations.

### Plant growth response in washed diesel-contaminated soil

**Fig. 8:**
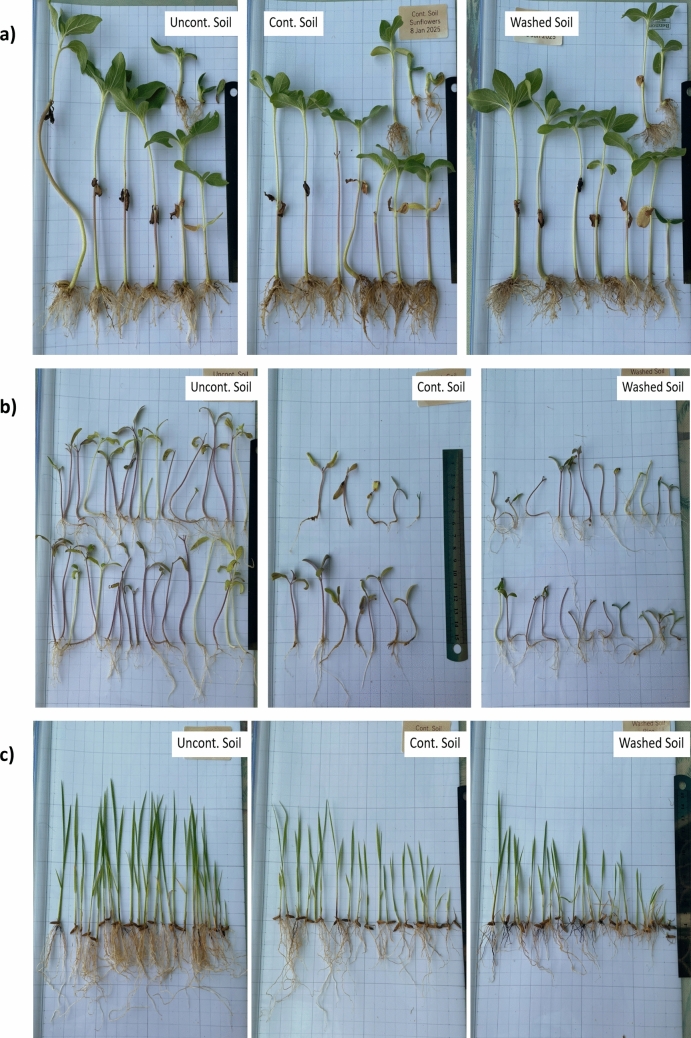
Seedlings grown in different soil treatments: **a** sunflower, **b** tomato, and **c** rice. Each species was cultivated in (1) clean control soil, (2) diesel-contaminated soil (20,000 mg/kg), and (3) washed soil treated with an optimized surfactant mixture

To evaluate the suitability of remediated soil for agricultural purposes, three plant species—sunflower, tomato, and rice—were cultivated in three different soil treatments: (1) uncontaminated clean soil (control), (2) diesel-contaminated soil (20,000 mg/kg), and (3) soil treated with a surfactant-based washing process optimized in earlier experiments. Visual assessment of seedling vigor (Fig. 8a–c) indicated that plants grown in control soil exhibited the most robust growth, characterized by greater height and greener foliage compared to those in diesel-contaminated and washed soils.

**Fig. 9:**
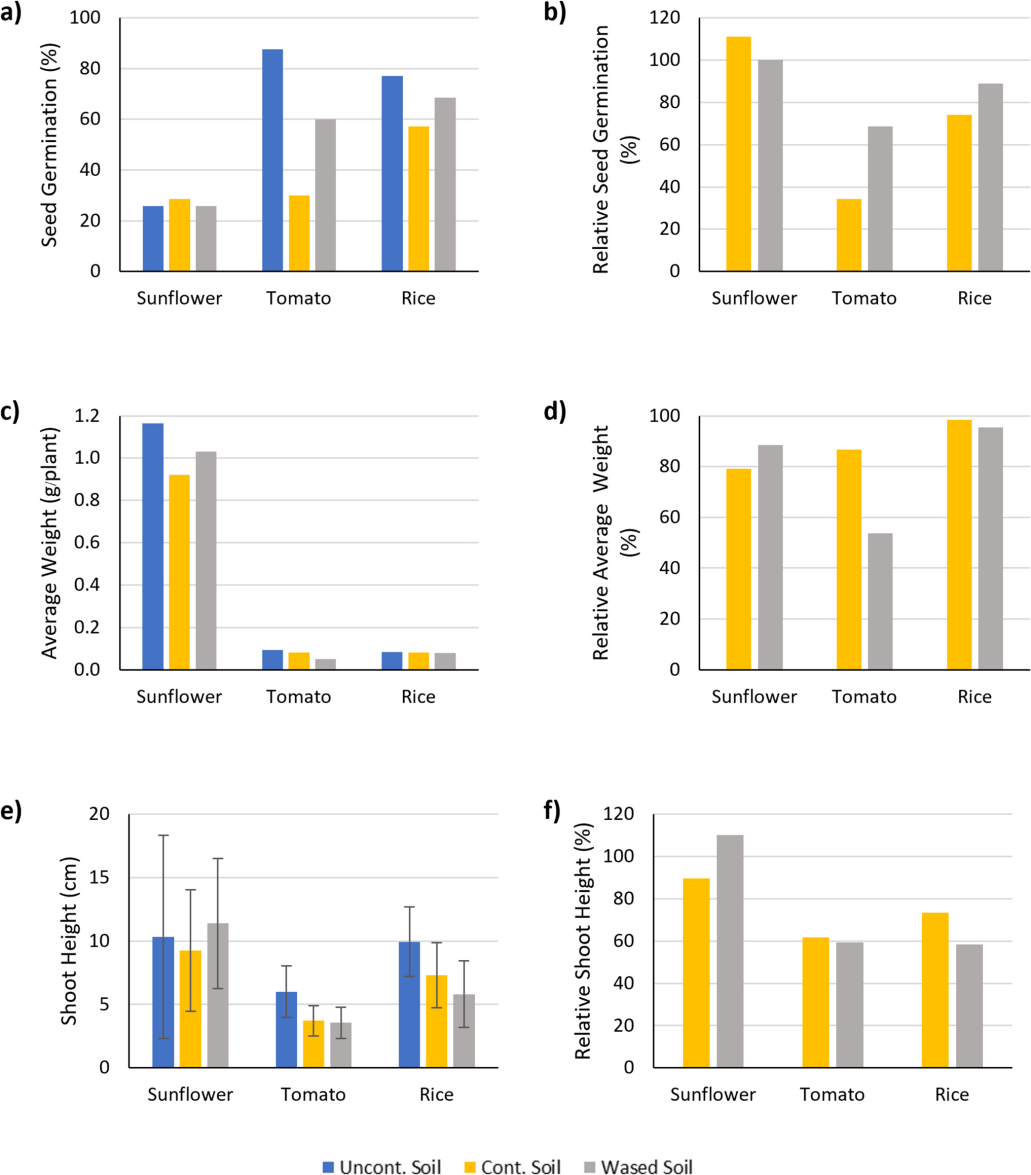
Germination performance and growth characteristics of sunflower, tomato, and rice cultivated in three soil treatments: uncontaminated soil (control), diesel-contaminated soil (cont. soil 20,000 mg/kg), and washed soil treated with an optimized surfactant mixture. **a** Seed germination, **b** relative seed germination compared to control, **c** average weight per plant, **d** relative weight compared to control, **e** average shoot height, and **f** relative shoot height compared to control

Germination rates varied among species and soil conditions. Sunflower seeds germinated consistently across all treatments, indicating higher tolerance to soil quality variations. In contrast, tomato seeds demonstrated marked sensitivity to contamination: germination was lowest in diesel-contaminated soil (30%), intermediate in washed soil (60%), and highest in control soil (87.5%). Rice seeds exhibited a similar pattern, though with less pronounced differences (Fig. 9a). The relative germination rate—calculated as a percentage of the control—clearly underscored the phytotoxic effects of diesel and the partial recovery facilitated by soil washing (Fig. 9b).

Average biomass per plant followed a similar trend, with control soil supporting the highest biomass across all species (Fig. 9c). For sunflower, biomass in washed soil reached 88.5% of the control, whereas diesel-contaminated soil resulted in 79.1% (Fig. 9d). However, for tomato and rice, biomass in washed soil was unexpectedly lower than in diesel-contaminated soil. This outcome may be attributed to nutrient loss caused by the washing process, particularly affecting species with higher nutrient demands.

Plant height data supported these findings (Fig. 9e, f). Sunflower height was greater in washed soil than in contaminated soil, suggesting a reduction in diesel toxicity. However, tomato and rice heights in washed soil were not significantly improved and, in some cases, slightly reduced. These results suggest that while the surfactant washing process effectively mitigates phytotoxicity, it may also deplete essential soil nutrients, thus limiting sustained plant development.

Supporting evidence from post-wash soil analysis (Table 4) revealed a reduction in clay content and a noticeable decline in key macronutrients, including potassium (K) and phosphorus (P). No detectable nitrogen (N) was found in any soil samples, consistent with the inherently low fertility of sandy loam soils. The removal of diesel likely facilitated nutrient leaching, resulting in diminished soil fertility. Interestingly, diesel-contaminated soils exhibited the highest organic matter and carbon content, likely due to residual hydrocarbons. Washed soils retained slightly elevated organic matter relative to the control, potentially due to incomplete diesel removal.

**Table 4:** Selected physico-chemical properties of clean (uncontaminated) soil, diesel-contaminated soil (20,000 mg/kg), and washed soil treated with an optimized surfactant mixture

Soil properties	Uncont. soil	Cont. soil	Washed soil
Soil texture	Loamy sand	Loamy sand	Loamy sand
Sand (%)	84.82	85.50	85.24
Silt (%)	7.27	6.60	10.85
Clay (%)	7.91	7.90	3.92
Carbon (%)	0.24 ± 0.03	0.53 ± 0.02	0.30 ± 0.04
Organic matter (%)	0.08 ± 0.05	0.61 ± 0.13	0.17 ± 0.08
Total N (mg/kg)	ND	ND	ND
Total K (mg/kg)	328.43 ± 62.62	363.11 ± 65.58	210.07 ± 10.81
Available P (mg/kg)	0.24 ± 0.07	0.18 ± 0.03	0.10 ± 0.02

*ND* not detectable

An incidental observation during tomato cultivation on day 28 revealed the presence of insect larvae feeding on the leaves of plants in washed soil. While this defoliation may have contributed to reduced tomato biomass, the presence of these organisms can also be interpreted as an indirect indicator of improved ecological compatibility, suggesting that the washed soil environment may be less toxic and capable of supporting early-stage ecological recovery.

Most notably, washed soil consistently yielded higher germination rates than diesel-contaminated soil, with relative germination rates of 70% for tomato and 90% for rice—values considered acceptable for preliminary ecological restoration efforts. These findings indicate that the applied biosurfactant-based washing process can substantially reduce soil toxicity to levels conducive to plant germination. However, the observed nutrient depletion highlights the need for subsequent soil amendment or fertilization to support complete plant development.

These results are consistent with Boonyathai (2018), who reported that combining biosurfactants with nonionic surfactants can reduce the overall toxicity of treated soils. While the present study provides promising evidence for the use of biosurfactants derived from water hyacinth in diesel-contaminated soil remediation, it also underscores the necessity for further investigation into nutrient management post-washing. Future studies should prioritize field-scale validation and consider long-term plant performance under varying fertilization regimes.

This research not only advances scientific understanding of phytotoxicity remediation but also contributes to the practical implementation of circular bioeconomy principles by valorizing an invasive aquatic plant species. Through the production and application of plant-based surfactants, this approach offers an environmentally sustainable and economically viable alternative for soil decontamination.

## Conclusion

This study demonstrates the potential of utilizing water hyacinth extract as a biosurfactant for the remediation of diesel-contaminated soils through soil washing. The extract exhibited a critical micelle concentration (CMC) of 0.9443% w/v and showed favorable surface-active properties. When combined with the synthetic surfactant Dehydol LS9, the formulated mixture enhanced emulsification and wettability, as evidenced by contact angle analysis on diesel-coated glass surfaces. These characteristics contributed to a diesel removal efficiency of 73.50% ± 5.65%, highlighting the effectiveness of the optimized formulation.

Phytotoxicity assays using sunflower, tomato, and rice seeds revealed that soil washing substantially improved germination rates compared to untreated contaminated soil, indicating a reduction in acute toxicity. However, plant biomass and height remained lower than those observed in uncontaminated control soils, suggesting that essential nutrients may have been lost during the washing process. Soil analysis supported this hypothesis, showing reduced levels of potassium, phosphorus, and clay content in post-wash soils.

These findings underscore both the environmental compatibility and remediation efficacy of the biosurfactant-based approach. The study provides scientific and practical support for integrating biosurfactants into sustainable soil remediation strategies, particularly by valorizing invasive aquatic plants such as water hyacinth within a circular bioeconomy framework. To further develop this approach for real-world applications, additional research is recommended at the pilot or field scale. Future studies should focus on designing in situ soil washing systems tailored to varying soil textures and contamination levels. Moreover, ecological impact assessments on soil biota, including microorganisms and earthworms, should be conducted to ensure long-term environmental safety. Enhancing post-treatment soil fertility through the application of biofertilizers or appropriate organic amendments is also advisable to promote ecological restoration and sustainable land use following remediation.

## Supplementary information

Below is the link to the electronic supplementary material.

ESM1 (DOCX 3.79 MB)

## Data Availability

The datasets used and analyzed during the current study are available from the corresponding author on reasonable request.
