# Supplementary material for: Ultrasound-assisted extraction of biosurfactants from water hyacinth for enhanced soil washing of diesel-contaminated soils: performance evaluation and phytotoxicity assessment
Source: Environ Sci Pollut Res Int. 2025 Sep 9;32(36):21522–42. doi: 10.1007/s11356-025-36930-2 (PMC12484369; doi:10.1007/s11356-025-36930-2)
Supplement: Supplementary file 1 — (DOCX 3.79 MB) [file 11356_2025_36930_MOESM1_ESM.docx]

**Ultrasound-Assisted Extraction of Biosurfactants from Water Hyacinth for Enhanced Soil Washing of Diesel-Contaminated Soils: Performance Evaluation and Phytotoxicity Assessment**

**Witchaya Rongsayamanont^a^ and Naphatsarnan Phasukarratchai^a^***

^a^Faculty of Environment and Resource Studies, Mahidol University, Salaya, Phutthamonthon, Nakhon Pathom, 73170, Thailand

*Corresponding author. E-mail: [naphatsarnan.pha@mahidol.ac.th](mailto:naphatsarnan.pha@mahidol.ac.th)

**Supplementary Information**

**1. Extract WH composition analysis by GCMS**

**
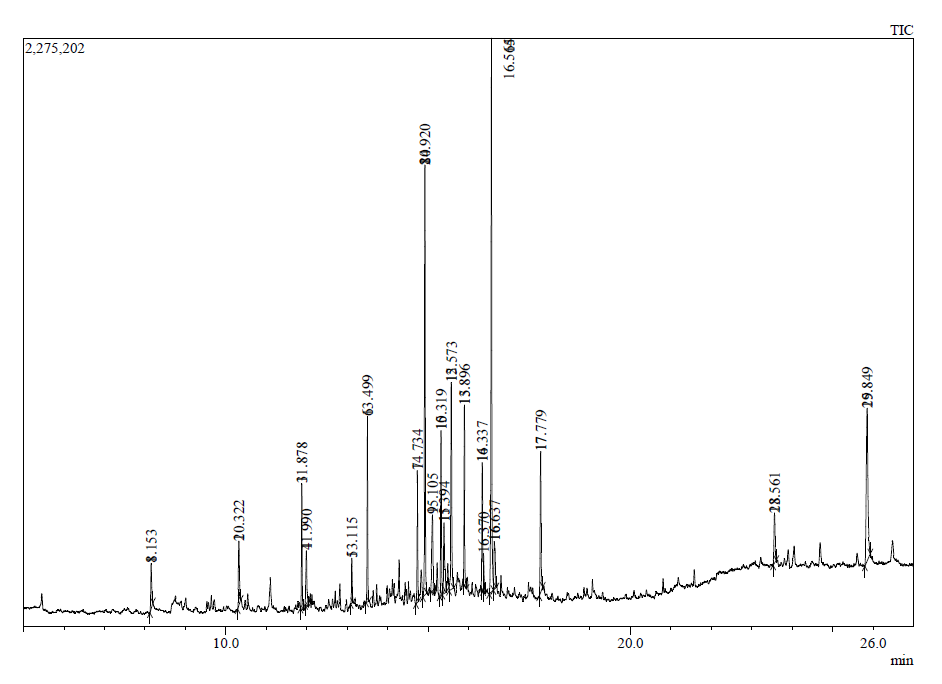
**

**Fig.S1** GCMS chromatogram of water-soluble extract WH

**
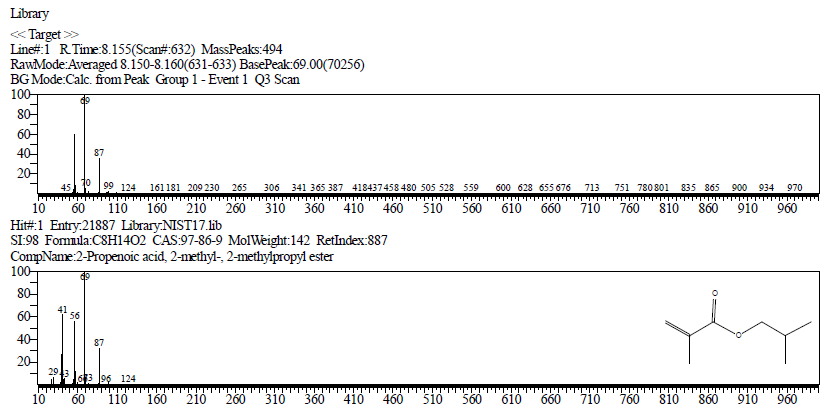
**

**Fig.S2** GCMS of the water-soluble extract WH compounds compared with reference libraries at RT 8.155 min


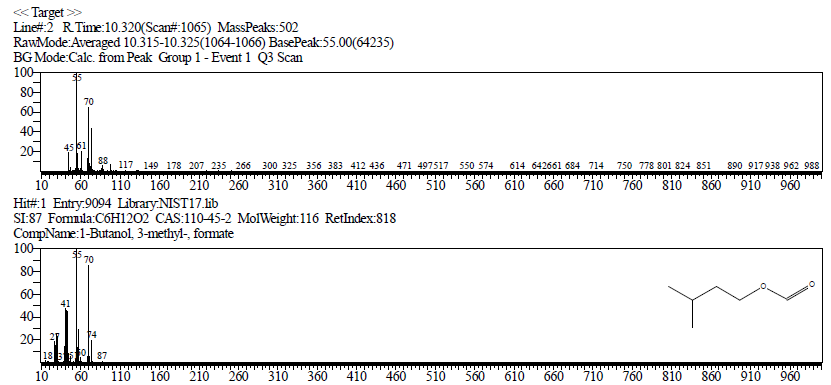


**Fig.S3** GCMS of the water-soluble extract WH compounds compared with reference libraries at RT 10.320 min


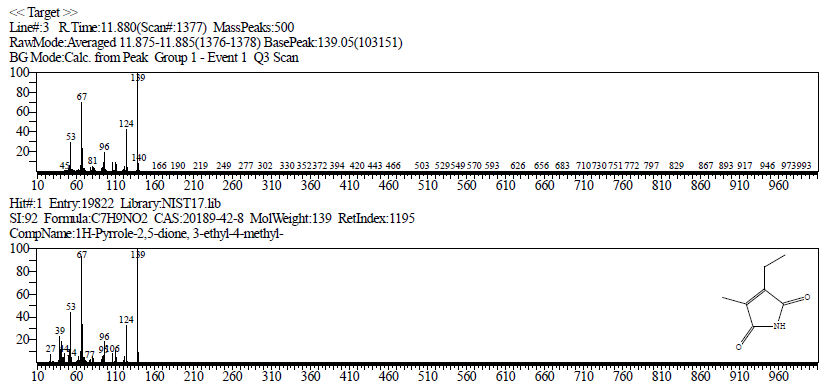


**Fig.S4** GCMS of the water-soluble extract WH compounds compared with reference libraries at RT 11.880 min


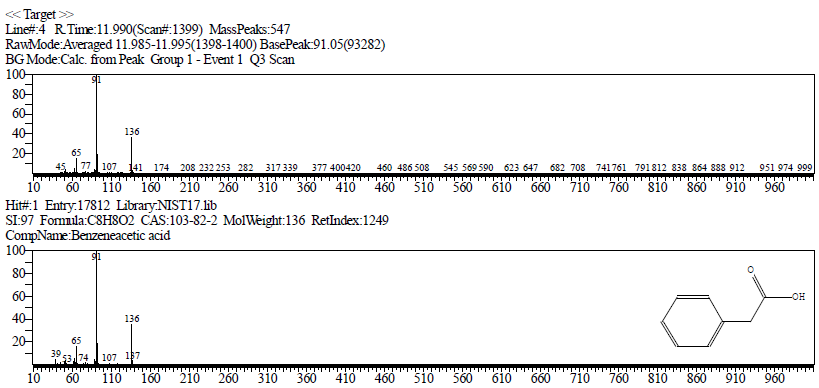


**Fig.S5** GCMS of the water-soluble extract WH compounds compared with reference libraries at RT 11.990 min


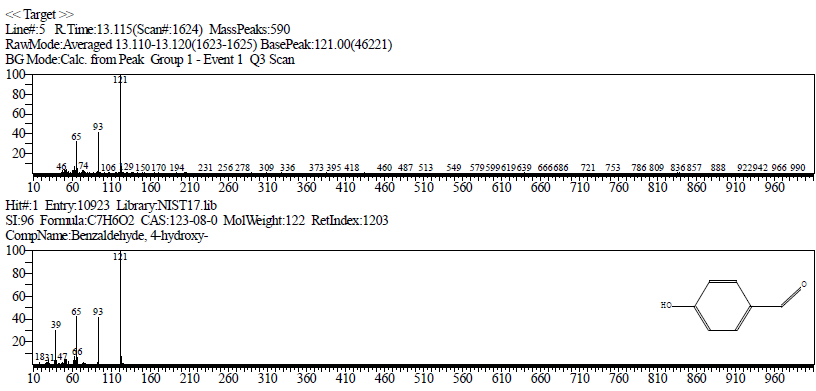


**Fig.S6** GCMS of the water-soluble extract WH compounds compared with reference libraries at RT 13.115 min


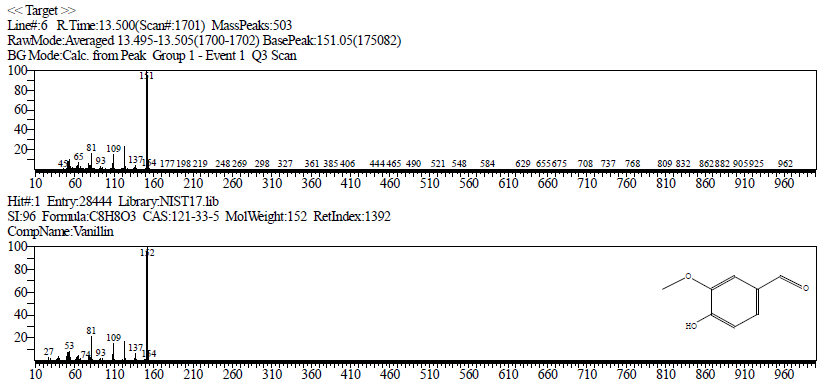


**Fig.S7** GCMS of the water-soluble extract WH compounds compared with reference libraries at RT 13.500 min


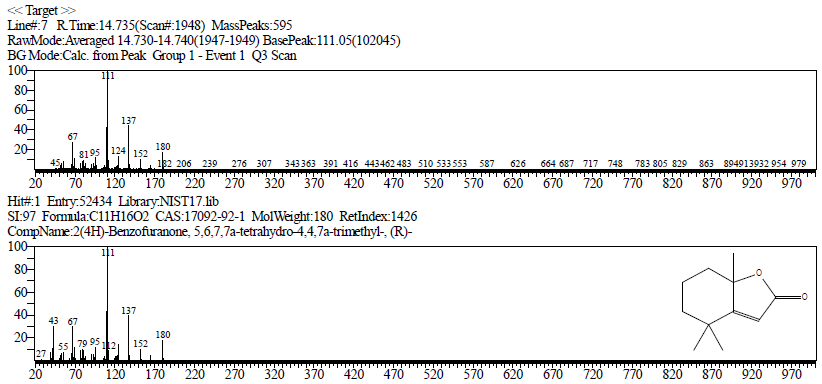


**Fig.S8** GCMS of the water-soluble extract WH compounds compared with reference libraries at RT 14.735 min


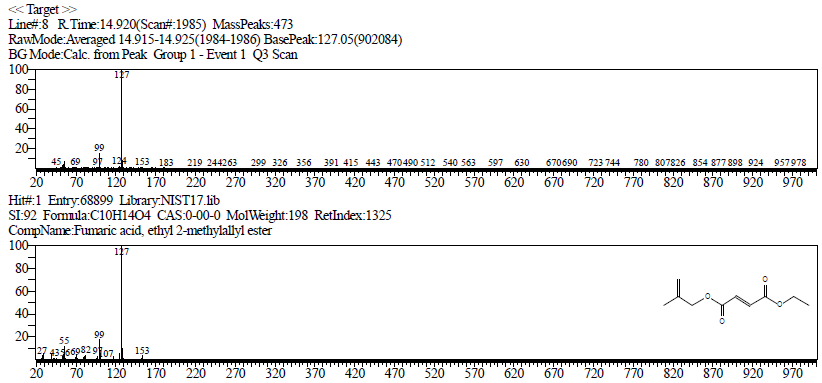


**Fig.S9** GCMS of the water-soluble extract WH compounds compared with reference libraries at RT 14.920 min


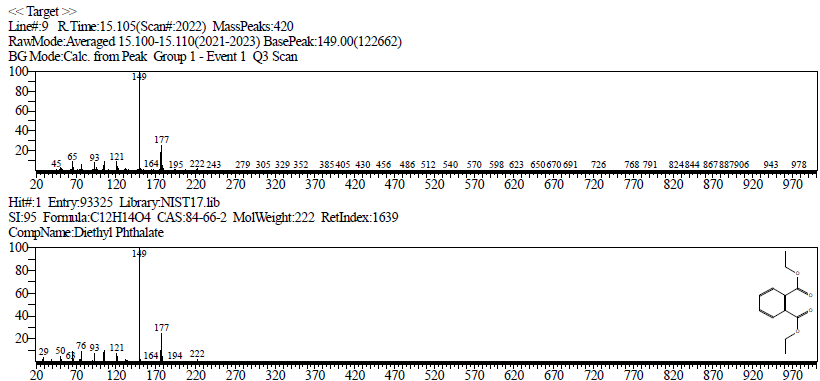


**Fig.S10** GCMS of the water-soluble extract WH compounds compared with reference libraries at RT15.105 min


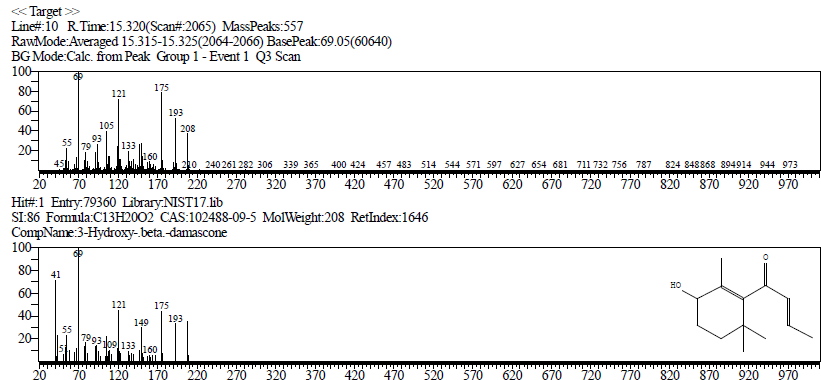


**Fig.S11** GCMS of the water-soluble extract WH compounds compared with reference libraries at RT 15.320 min


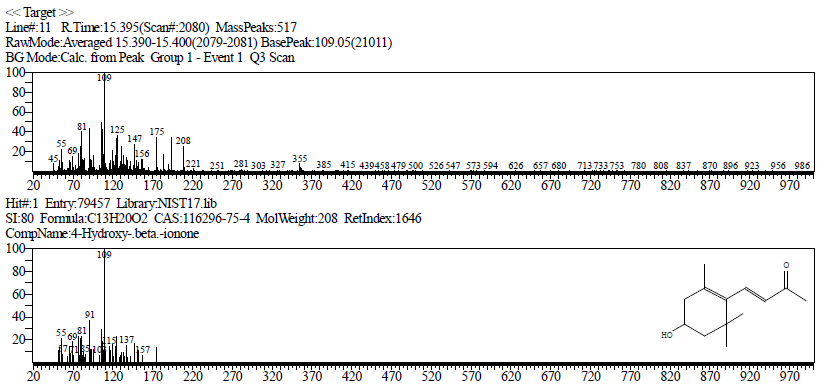


**Fig.S12** GCMS of the water-soluble extract WH compounds compared with reference libraries at RT 15.395 min


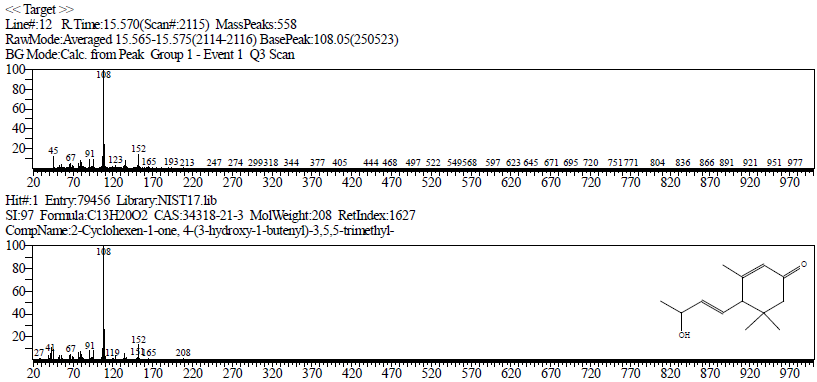


**Fig.S13** GCMS of the water-soluble extract WH compounds compared with reference libraries at RT 15.570 min


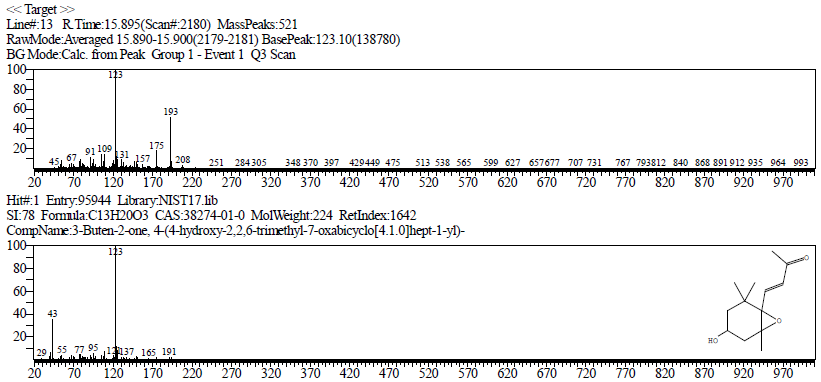


**Fig.S14** GCMS of the water-soluble extract WH compounds compared with reference libraries at RT 15.895 min


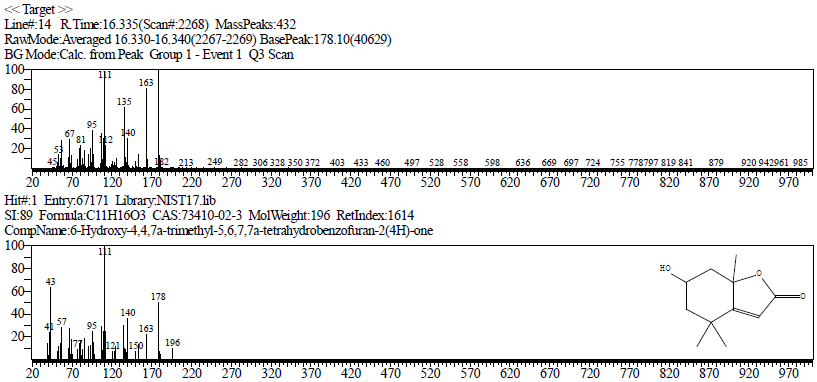


**Fig.S15** GCMS of the water-soluble extract WH compounds compared with reference libraries at RT 16.335 min


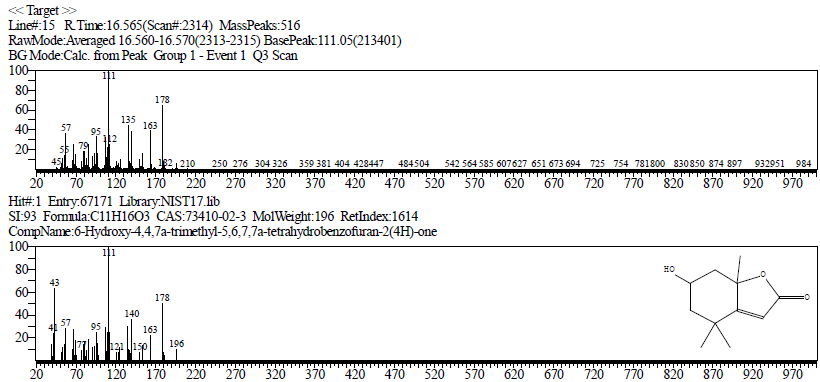


**Fig.S16** GCMS of the water-soluble extract WH compounds compared with reference libraries at RT 16.565 min


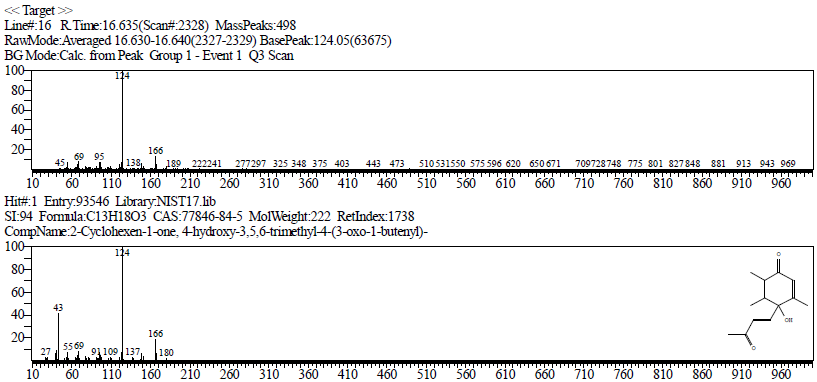


**Fig.S17** GCMS of the water-soluble extract WH compounds compared with reference libraries at RT 16.635 min


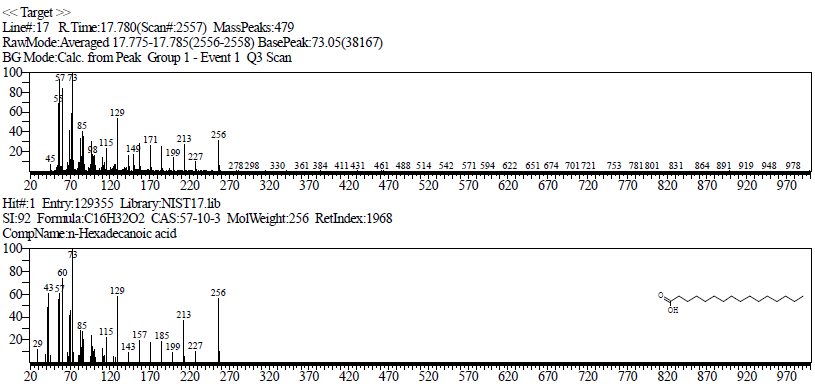


**Fig.S18** GCMS of the water-soluble extract WH compounds compared with reference libraries at RT 17.780 min


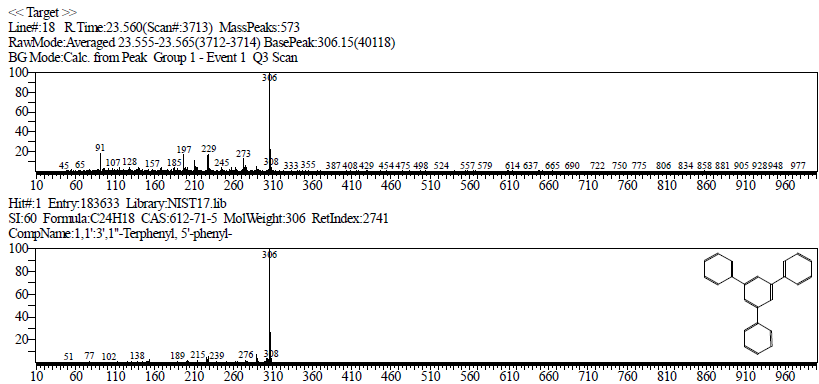


**Fig.S19** GCMS of the water-soluble extract WH compounds compared with reference libraries at RT 23.560 min


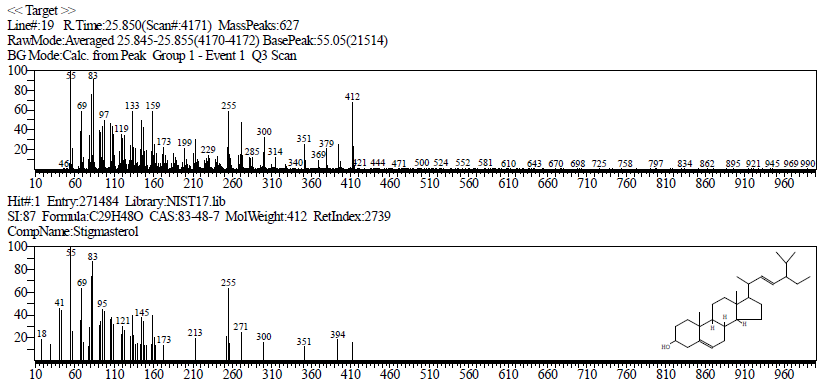


**Fig.S20** GCMS of the water-soluble extract WH compounds compared with reference libraries at RT 25.850 min

**2. Extract WH composition analysis by LCMSMS**

**
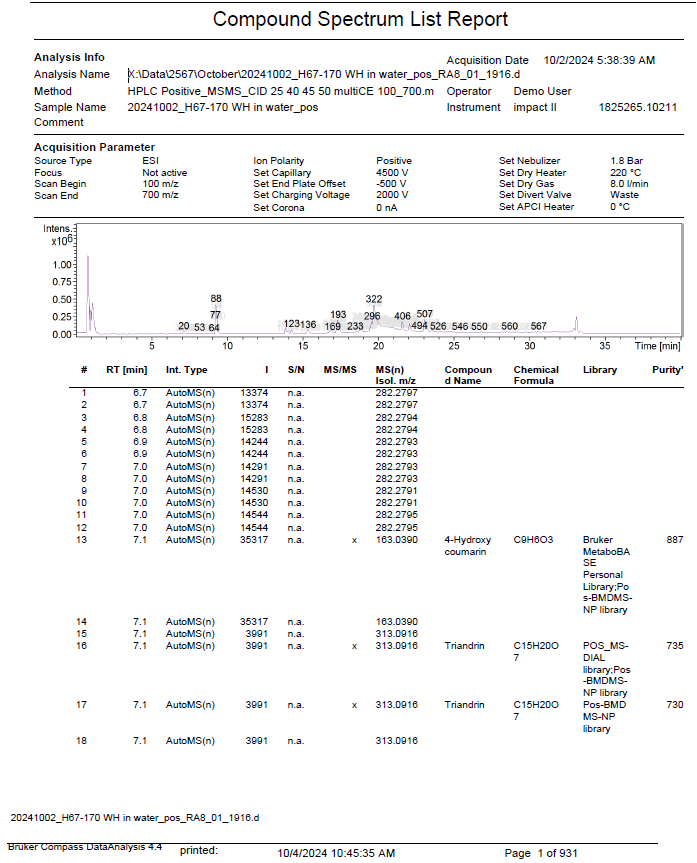
**

**
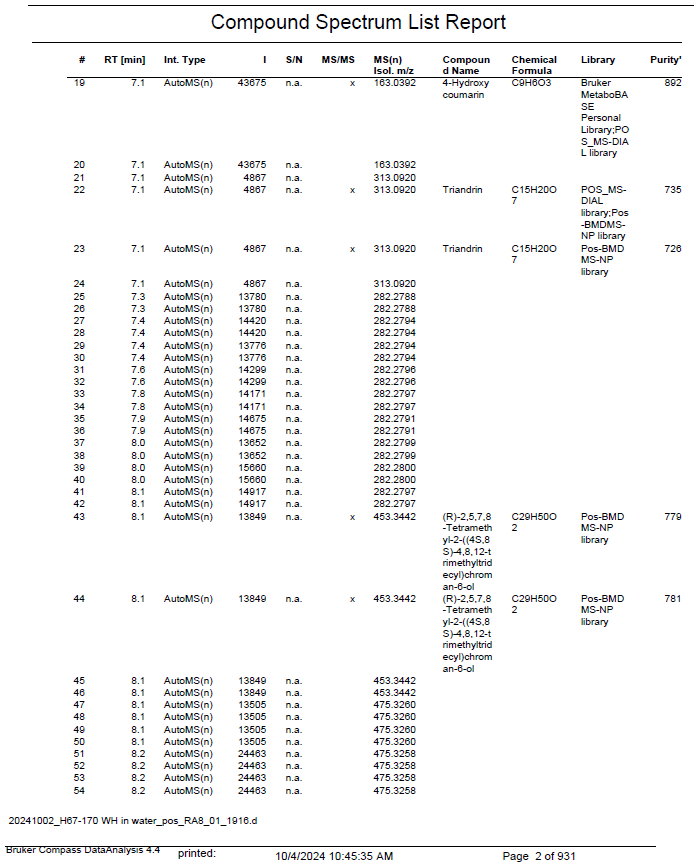

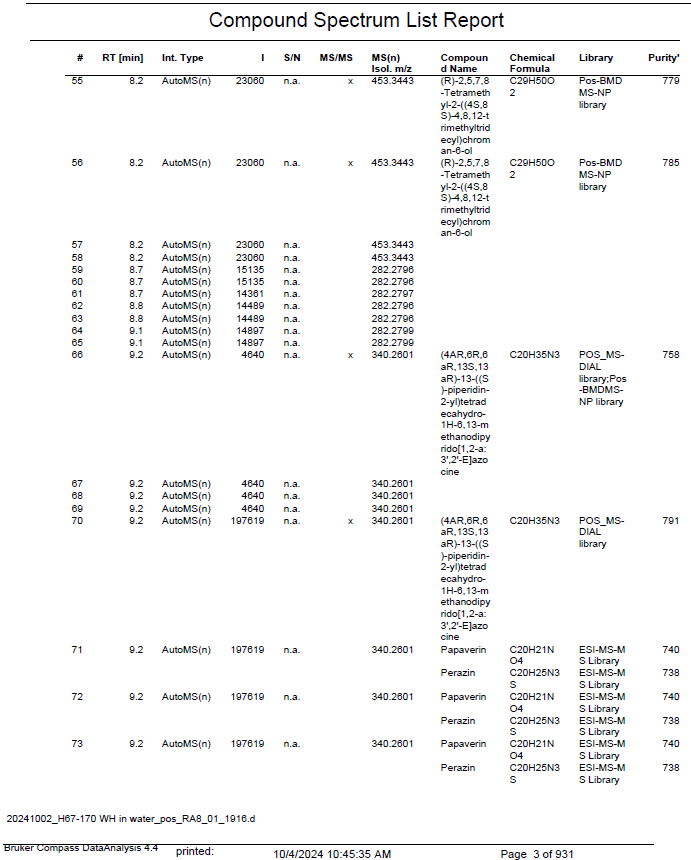

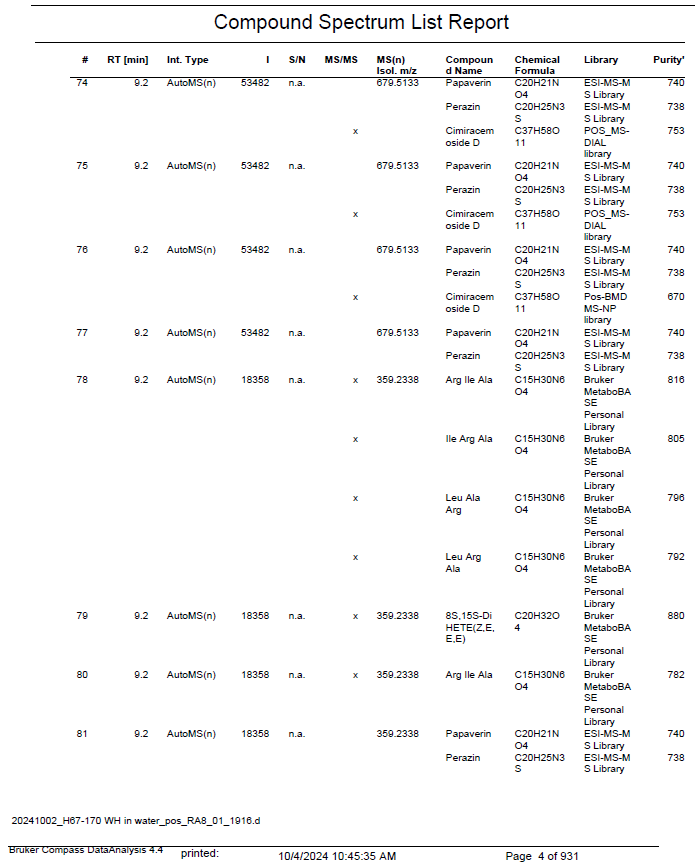

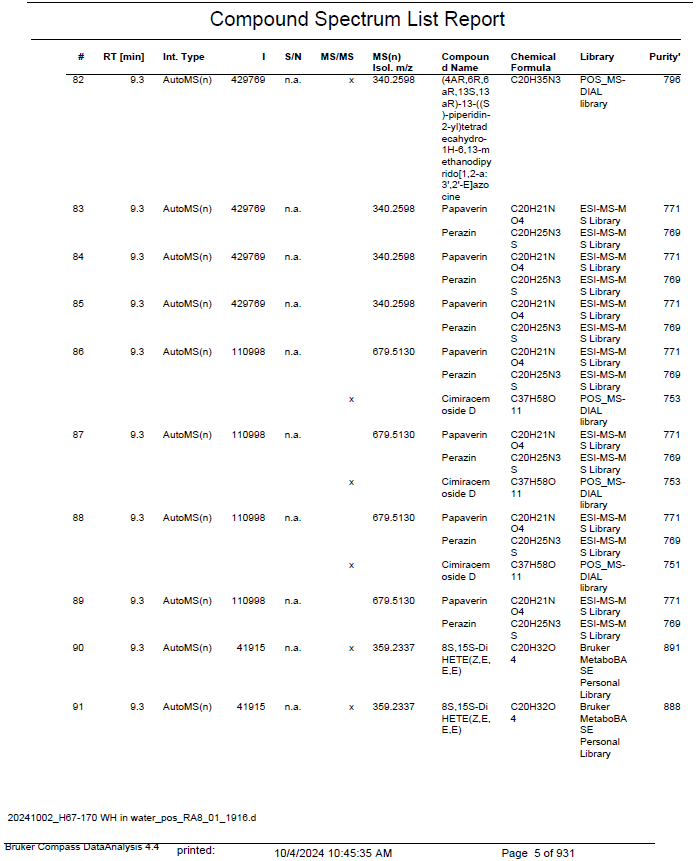

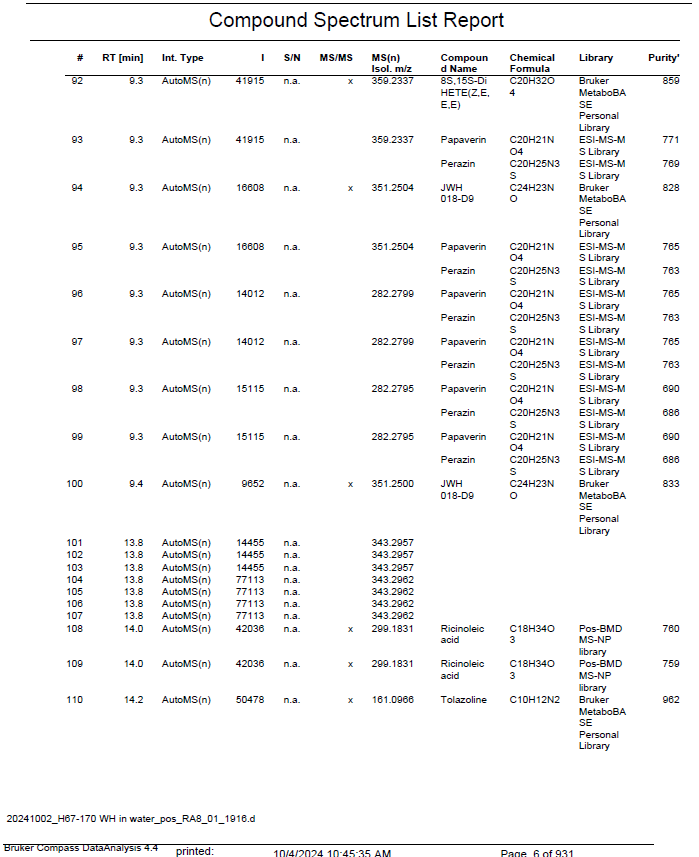

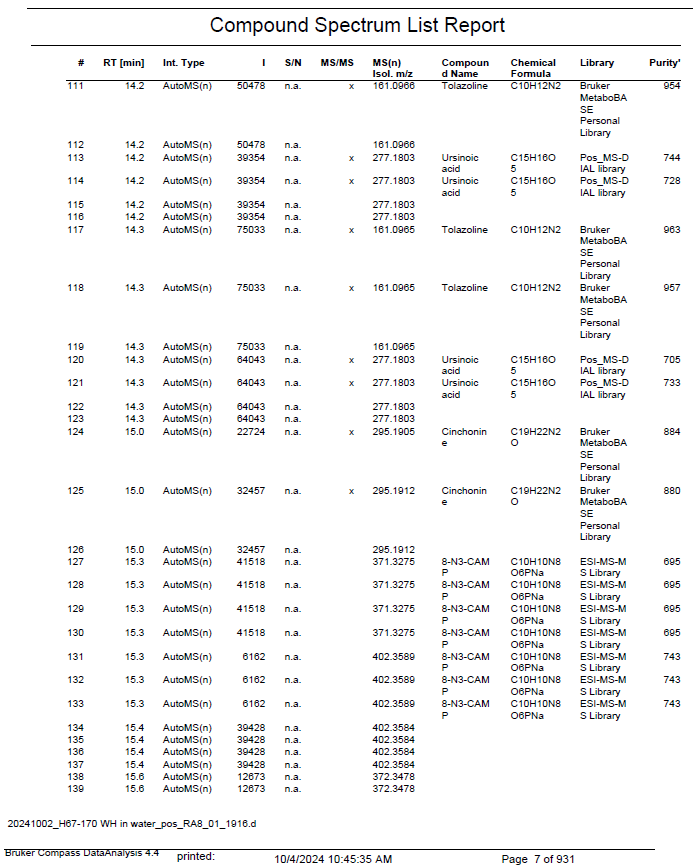

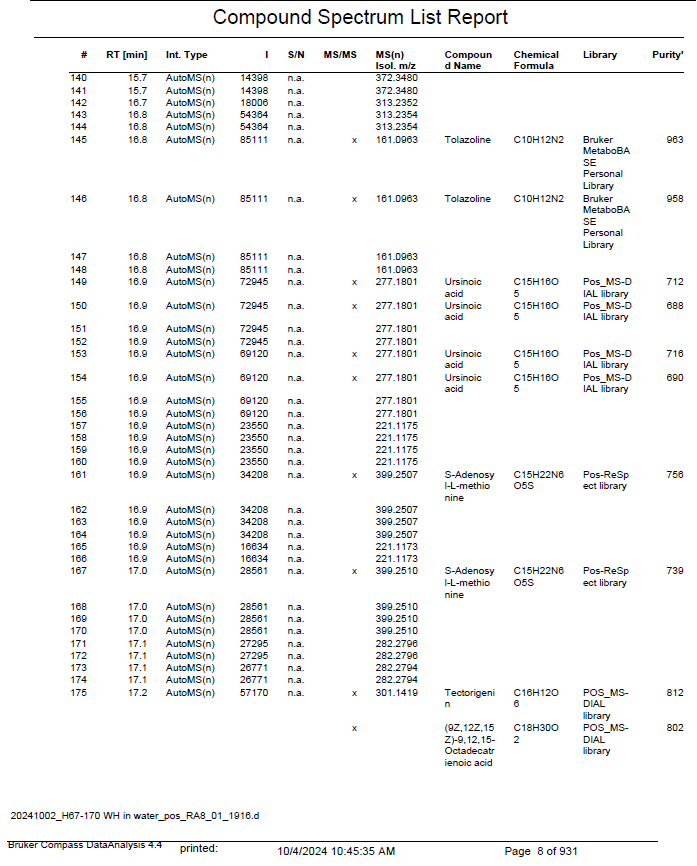

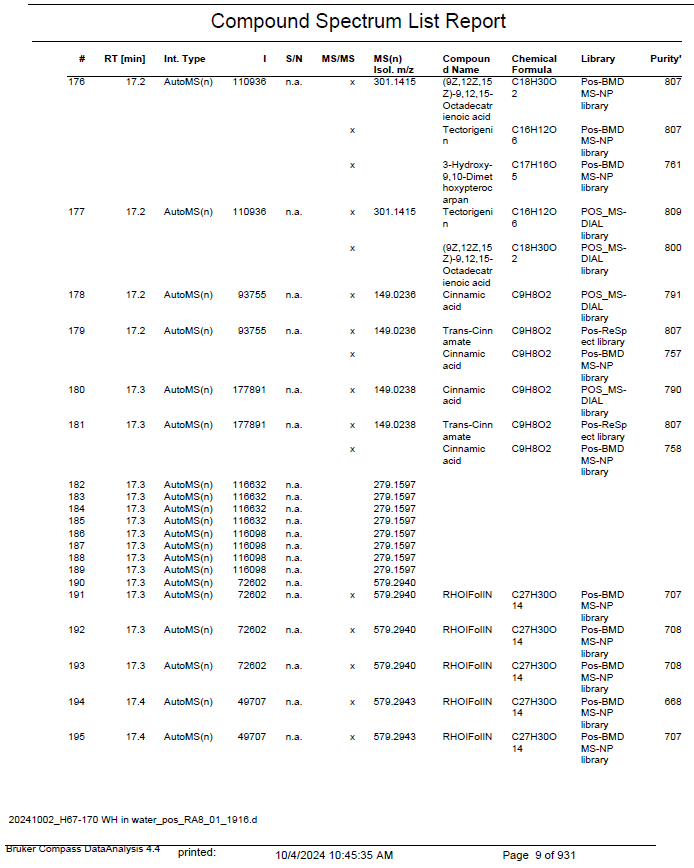

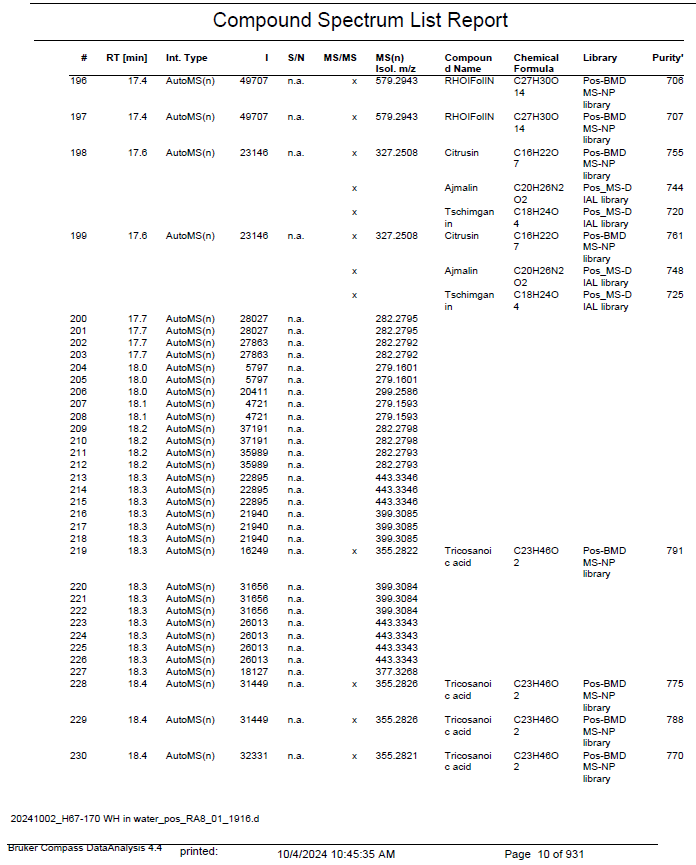

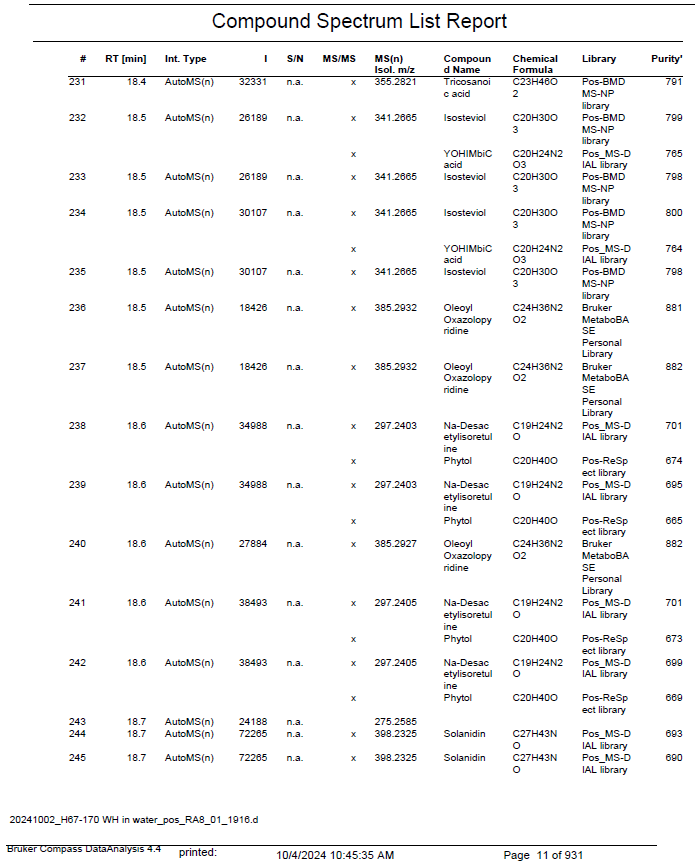

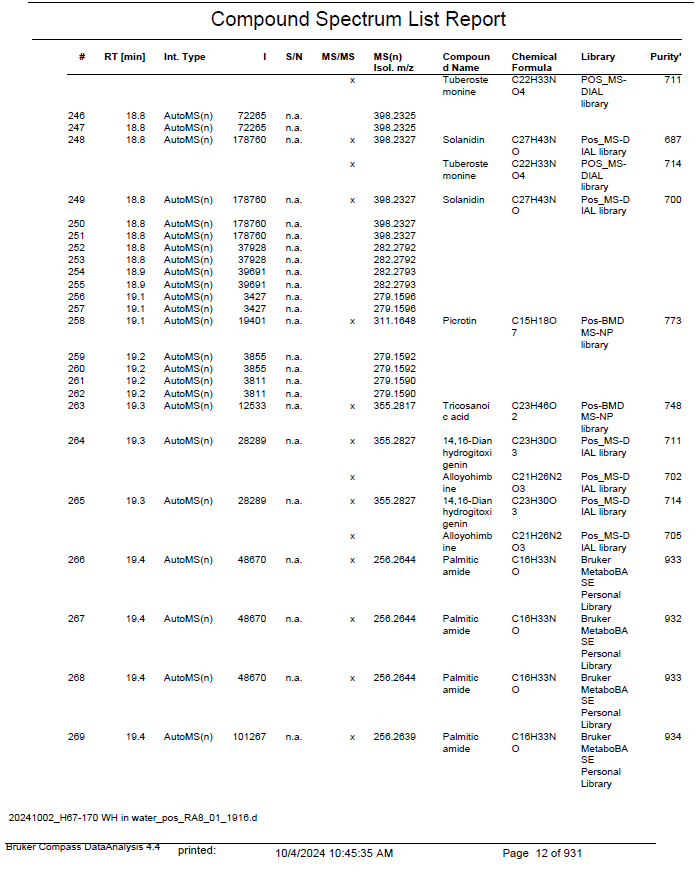

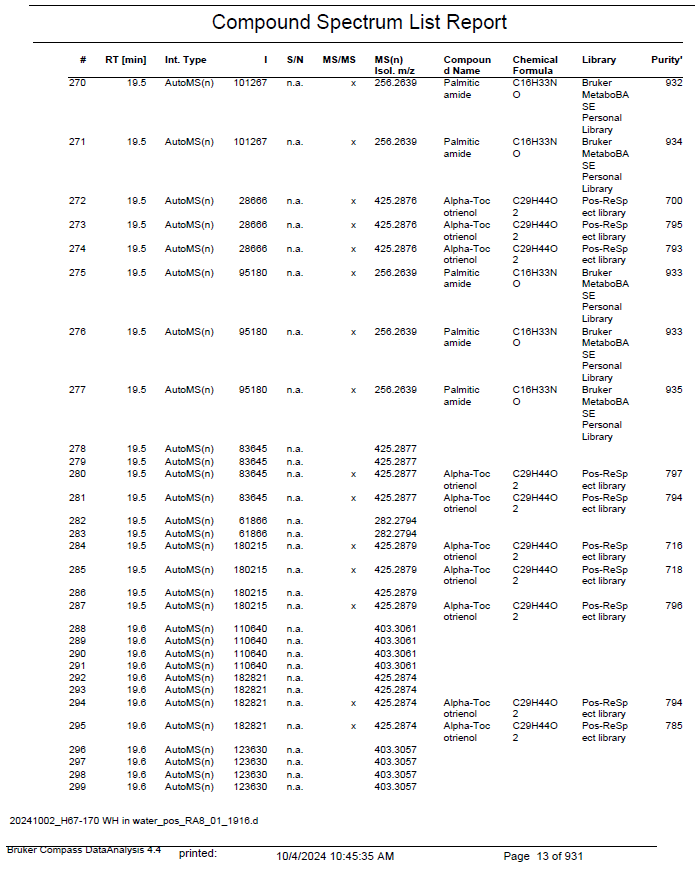

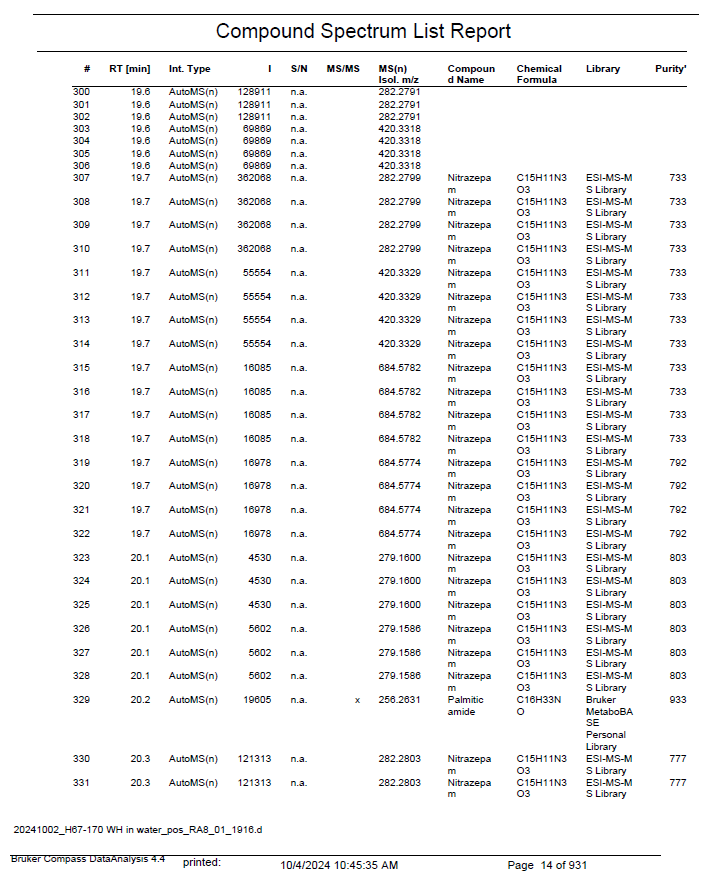

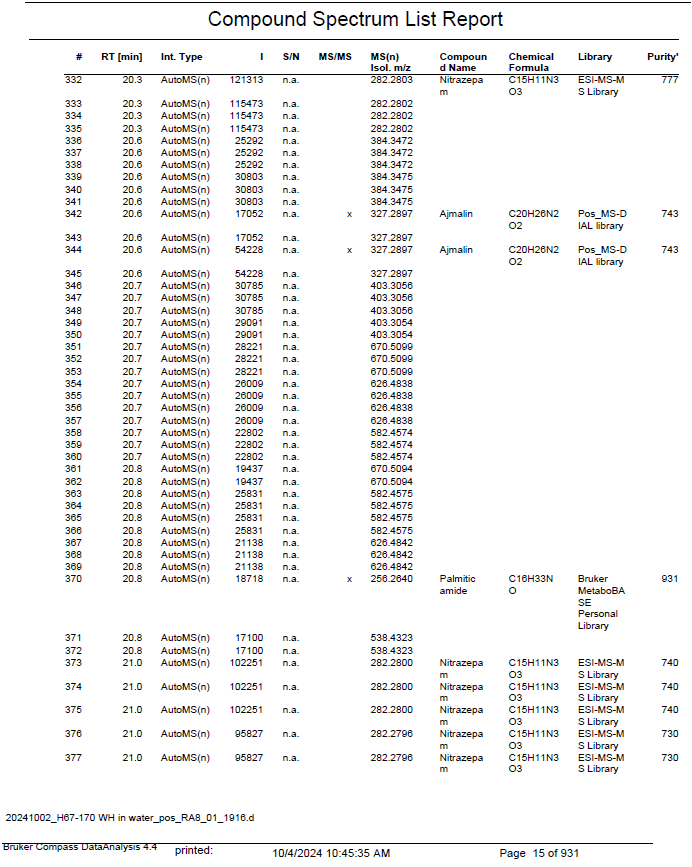

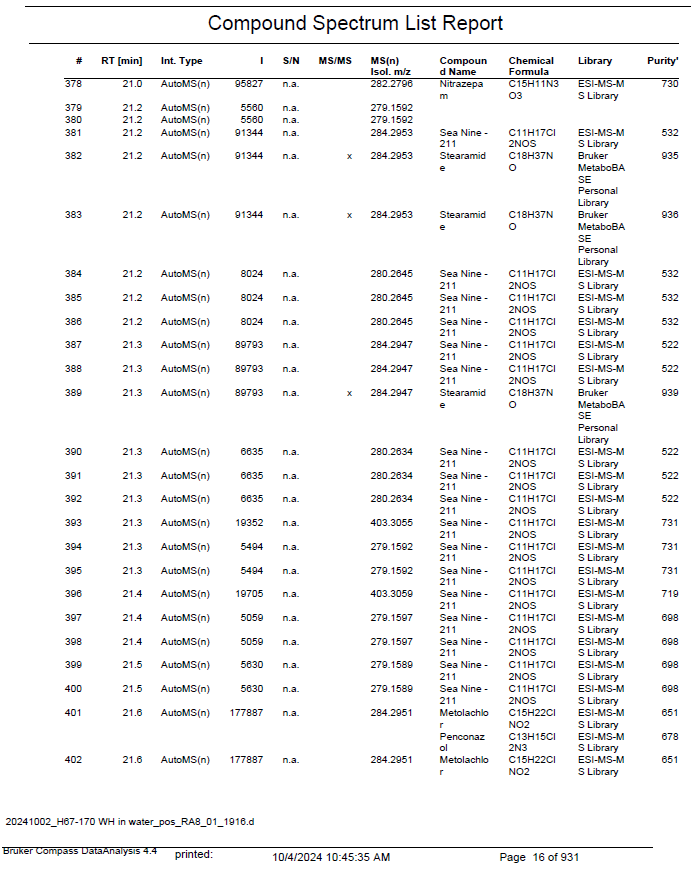

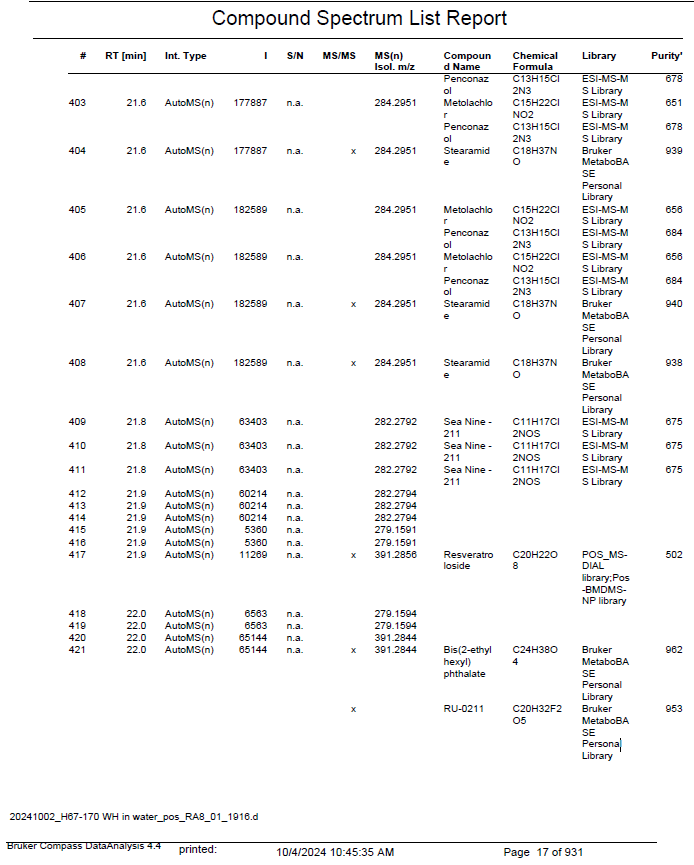

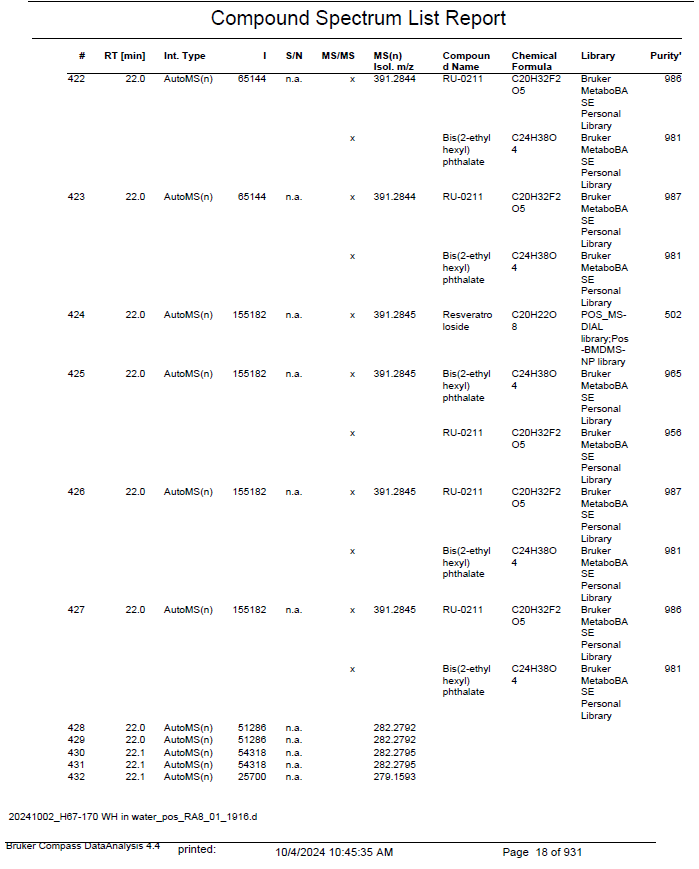

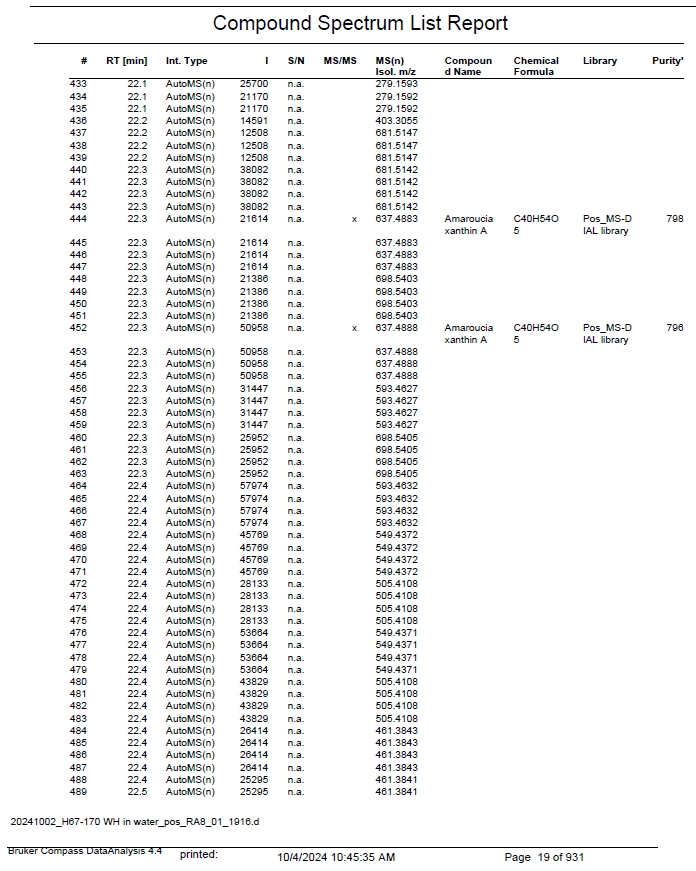

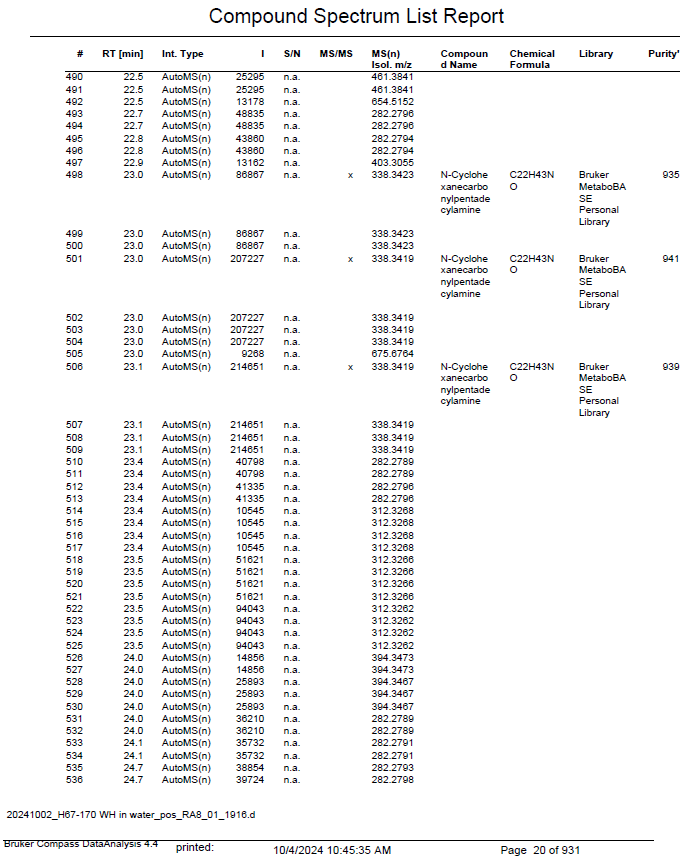

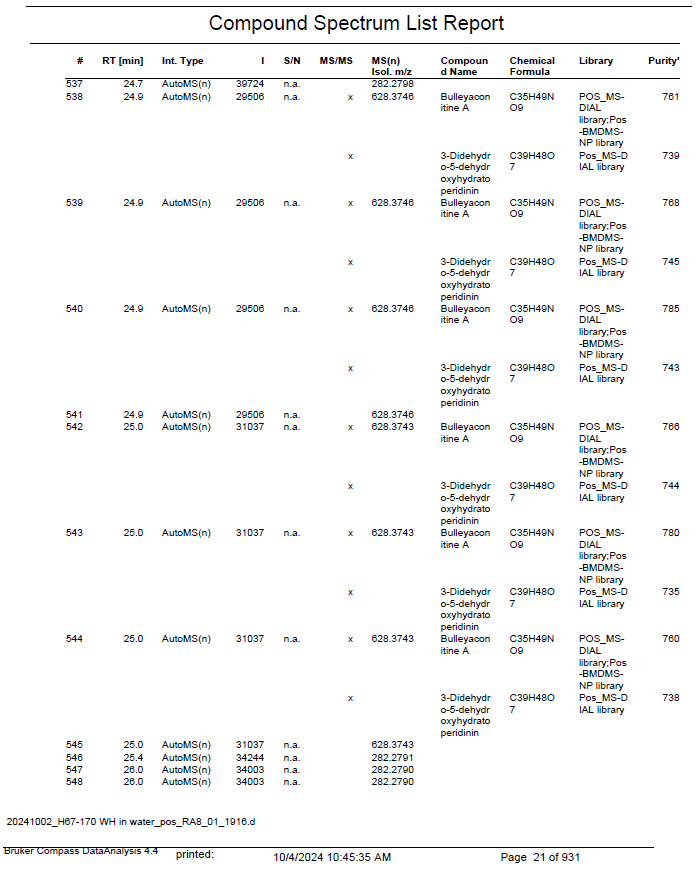

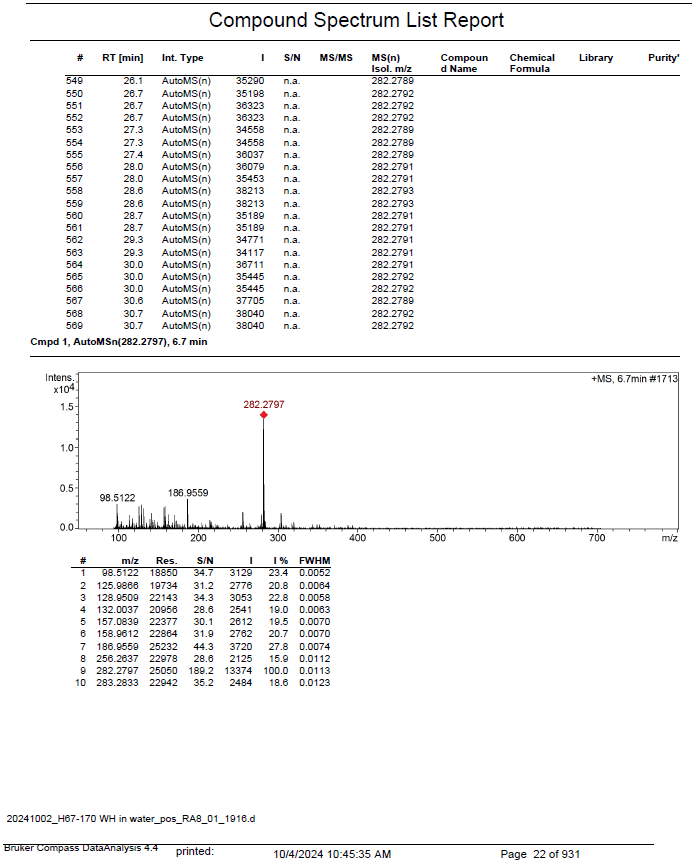
**

**3. Mixture Design Results**

**Table S1** Diesel-contaminated soil washing efficiency using 2% mixed surfactant solutions at various mixing ratios

| Sample Run | Washing solution concentration | | | Diesel removal (%) | | | | |
| --- | --- | --- | --- | --- | --- | --- | --- | --- |
|  | Dehydol LS9 (%) | Extract WH (%) | NaCl (%) | Rep 1 | Rep 2 | AVG | SD | %RSD |
| 1 | 2.00 | 0.00 | 0.00 | 80.53 | 83.93 | 82.23 ^e^ | 2.40 | 2.92 |
| 2 | 0.00 | 2.00 | 0.00 | 32.58 | 41.33 | 36.96 ^a^ | 6.19 | 16.75 |
| 3 | 0.00 | 0.00 | 2.00 | 51.08 | 51.73 | 51.40 ^b^ | 0.46 | 0.89 |
| 4 | 1.00 | 1.00 | 0.00 | 71.28 | 59.30 | 65.29 ^c^ | 8.47 | 12.98 |
| 5 | 1.00 | 0.00 | 1.00 | 75.01 | 66.41 | 70.71 ^c,d^ | 6.08 | 8.60 |
| 6 | 0.00 | 1.00 | 1.00 | 36.74 | 46.69 | 41.72 ^a,b^ | 7.03 | 16.86 |
| 7 | 1.33 | 0.33 | 0.33 | 69.13 | 66.19 | 67.66 ^c^ | 2.08 | 3.07 |
| 8 | 0.33 | 1.33 | 0.33 | 65.48 | 71.77 | 68.63 ^c^ | 4.45 | 6.49 |
| 9 | 0.33 | 0.33 | 1.33 | 72.10 | 71.25 | 71.67 ^c,d,e^ | 0.60 | 0.83 |
| 10 | 0.67 | 0.67 | 0.67 | 80.33 | 81.54 | 80.94 ^d,e^ | 0.86 | 1.06 |
| DI water | - | - | - | 7.59 | 9.86 | 8.73 | 1.61 | 18.43 |

**Note:** a, b, c, d, and e indicate statistically significant differences based on ANOVA at *p* = 0.05.

**Table S2** Statistical analysis results of diesel removal efficiency using surfactant mixtures from the mixture design, expressed in the form of a regression equation

| Model | F | p | R-Sqr | Adjust R-Sqr |
| --- | --- | --- | --- | --- |
| Linear | 10.06936 | 0.001304 | 0.542257 | 0.488404 |
| Quadratic | 1.90026 | 0.175983 | 0.674713 | 0.558539 |
| Special Cubic | 9.12748 | 0.009830 | 0.808892 | 0.720689 |
| Cubic | 15.31165 | 0.000663 | 0.949495 | 0.912764 |
| Total Adjusted |  |  |  |  |

**Table S3** Model adequacy analysis of the cubic regression equation for predicting diesel removal efficiency using surfactant mixtures from the mixture design

|  | SS | df | MS | F | p |
| --- | --- | --- | --- | --- | --- |
| Model | 4290.123 | 8 | 536.2654 | 25.85006 | 0.000004 |
| Total Error | 228.198 | 11 | 20.7452 | - | - |
| Lack of Fit | 0.438 | 1 | 0.4383 | 0.01925 | 0.892420 |
| Pure Error | 227.759 | 10 | 22.7759 | - | - |
| Total Adjusted | 4518.321 | 19 | 237.8064 | - | - |

The cubic regression equation for predicting diesel removal efficiency using surfactant mixtures from the mixture design is following equation.

v = 82.20x + 36.92y + 51.37z + 22.65xy + 15.45xz - 9.98yz + 558.55xyz - 175.77xy(x-y) - 111.42xz(x-z)

Here, v, x, y, and z represent diesel removal (%), Dehydol LS9 (%), Extract WH (%), and NaCl (%), respectively.

**4. Concentration and Time Optimization for soil washing**

**Table S4** Diesel removal efficiency in CCRD experiment

| **Sample Run** | **Conc. (%)** | **Time (min)** | **Diesel removal (%)** | | | |
| --- | --- | --- | --- | --- | --- | --- |
|  |  |  | **Rep1** | **Rep 2** | **AVG** | **SD** |
| 1 | 0.50 | 15.00 | 52.2 | 53.8 | 53.0 | 1.09 |
| 2 | 0.50 | 45.00 | 58.4 | 58.0 | 58.2 | 0.22 |
| 3 | 1.50 | 15.00 | 61.8 | 61.0 | 61.4 | 0.55 |
| 4 | 1.50 | 45.00 | 61.2 | 61.0 | 61.1 | 0.14 |
| 5 | 0.29 | 30.00 | 46.7 | 54.3 | 50.5 | 5.39 |
| 6 | 1.71 | 30.00 | 70.3 | 72.0 | 71.2 | 1.15 |
| 7 | 1.00 | 8.79 | 55.7 | 62.1 | 58.9 | 4.57 |
| 8 | 1.00 | 51.21 | 61.0 | 69.5 | 65.2 | 6.01 |
| 9 (C) | 1.00 | 30.00 | 80.3 | 68.9 | 74.6 | 8.08 |
| 10 (C) | 1.00 | 30.00 | 60.5 | 61.1 | 60.8 | 0.42 |

**Table S5** Model adequacy analysis of the regression equation for predicting diesel removal efficiency from CCRD
